# Association of diabetes, smoking, and alcohol use with subclinical-to-symptomatic spectrum of tuberculosis in 16 countries: an individual participant data meta-analysis of national tuberculosis prevalence surveys

**DOI:** 10.1016/j.eclinm.2023.102191

**Published:** 2023-08-30

**Authors:** Yohhei Hamada, Matteo Quartagno, Irwin Law, Farihah Malik, Frank Adae Bonsu, Ifedayo M.O. Adetifa, Yaw Adusi-Poku, Umberto D'Alessandro, Adedapo Olufemi Bashorun, Vikarunnessa Begum, Dina Bisara Lolong, Tsolmon Boldoo, Themba Dlamini, Simon Donkor, Bintari Dwihardiani, Saidi Egwaga, Muhammad N. Farid, Anna Marie Celina G.Garfin, Donna Mae G Gaviola, Mohammad Mushtuq Husain, Farzana Ismail, Mugagga Kaggwa, Deus V. Kamara, Samuel Kasozi, Kruger Kaswaswa, Bruce Kirenga, Eveline Klinkenberg, Zuweina Kondo, Adebola Lawanson, David Macheque, Ivan Manhiça, Llang Bridget Maama-Maime, Sayoki Mfinanga, Sizulu Moyo, James Mpunga, Thuli Mthiyane, Dyah Erti Mustikawati, Lindiwe Mvusi, Hoa Binh Nguyen, Hai Viet Nguyen, Lamria Pangaribuan, Philip Patrobas, Mahmudur Rahman, Mahbubur Rahman, Mohammed Sayeedur Rahman, Thato Raleting, Pandu Riono, Nunurai Ruswa, Elizeus Rutebemberwa, Mugabe Frank Rwabinumi, Mbazi Senkoro, Ahmad Raihan Sharif, Welile Sikhondze, Charalambos Sismanidis, Tugsdelger Sovd, Turyahabwe Stavia, Sabera Sultana, Oster Suriani, Albertina Martha Thomas, Kristina Tobing, Martie Van der Walt, Simon Walusimbi, Mohammad Mostafa Zaman, Katherine Floyd, Andrew Copas, Ibrahim Abubakar, Molebogeng X. Rangaka

**Affiliations:** a Institute for Global Health, University College London, United Kingdom; b MRC Clinical Trials Unit, Institute of Clinical Trials and Methodology, University College London, United Kingdom; c Global Tuberculosis Programme, World Health Organization, Switzerland; d UCL Great Ormond Street Institute of Child Health, University College London, United Kingdom; e National Tuberculosis Programme, Ghana Health Service, Ghana; f Disease Control and Elimination Theme, Medical Research Council Unit The Gambia at London School of Hygiene and Tropical Medicine, Gambia; g Department of Infectious Diseases Epidemiology, London School of Hygiene & Tropical Medicine, United Kingdom; h World Health Organization, Country Office for Bangladesh, Bangladesh; i National Research and Innovation Agency, Indonesia; j Tuberculosis Surveillance and Research Department, National Center for Communicable Disease, Mongolia; k Eswatini National Tuberculosis Program, Ministry of Health, Eswatini; l Center for Tropical Medicine, Faculty of Medicine, Public Health and Nursing, Gadjah Mada University, Indonesia; m Tuberculosis and Leprosy Programme, Ministry of Health and Social Welfare, United Republic of Tanzania; n Expert TB Committee, Indonesia; o Department of Health, Philippines; p Institute of Epidemiology, Disease Control and Research (IEDCR), Bangladesh; q Centre for Tuberculosis, National Institute for Communicable Diseases, A Division of the National Health Laboratory Services, South Africa; r Department of Medical Microbiology, University of Pretoria, South Africa; s World Health Organization, Country Office for Uganda, Uganda; t National Tuberculosis Control Programme, Ministry of Health, Uganda; u National Tuberculosis Programme, Ministry of Health, Malawi; v Makerere University Lung Institute, Uganda; w Department of Global Health, Amsterdam University Medical Center, Netherlands; x National Tuberculosis and Leprosy Control Programme, Federal Ministry of Health, Nigeria; y National Tuberculosis Program, Ministry of Health, Mozambique; z Ministry of Health TB and Leprosy Programme, Lesotho; aa National Institute for Medical Research, Muhimbili Medical Research Centre, United Republic of Tanzania; ab Liverpool School of Tropical Medicine, United Kingdom; ac Alliance for Africa Health and Research, United Republic of Tanzania; ad Human Sciences Research Council, South Africa; ae School of Public Health and Family Medicine, University of Cape Town, South Africa; af South African Medical Research Council, South Africa; ag Ministry of Health, Indonesia; ah National Department of Health, South Africa; ai National Tuberculosis Programme, Viet Nam; aj World Health Organization, Country Office for Nigeria, Nigeria; ak University of Indonesia, Indonesia; al Ministry of Health and Social Services, Namibia; am Department of Health Policy, Planning and Management, Makerere University School of Public Health, Uganda; an Ministry of Health, Mongolia; ao Division of Epidemiology and Biostatistics & CIDRI-AFRICA, University of Cape Town, South Africa

**Keywords:** NCD, Smoking: tobacco, TB, Diabetes, Screening

## Abstract

**Background:**

Non-communicable diseases (NCDs) and NCD risk factors, such as smoking, increase the risk for tuberculosis (TB). Data are scarce on the risk of prevalent TB associated with these factors in the context of population-wide systematic screening and on the association between NCDs and NCD risk factors with different manifestations of TB, where ∼50% being asymptomatic but bacteriologically positive (subclinical). We did an individual participant data (IPD) meta-analysis of national and sub-national TB prevalence surveys to synthesise the evidence on the risk of symptomatic and subclinical TB in people with NCDs or risk factors, which could help countries to plan screening activities.

**Methods:**

In this systematic review and IPD meta-analysis, we identified eligible prevalence surveys in low-income and middle-income countries that reported at least one NCD (e.g., diabetes) or NCD risk factor (e.g., smoking, alcohol use) through the archive maintained by the World Health Organization and by searching in Medline and Embase from January 1, 2000 to August 10, 2021. The search was updated on March 23, 2023. We performed a one-stage meta-analysis using multivariable multinomial models. We estimated the proportion of and the odds ratio for subclinical and symptomatic TB compared to people without TB for current smoking, alcohol use, and self-reported diabetes, adjusted for age and gender. Subclinical TB was defined as microbiologically confirmed TB without symptoms of current cough, fever, night sweats, or weight loss and symptomatic TB with at least one of these symptoms. We assessed heterogeneity using forest plots and I^2^ statistic. Missing variables were imputed through multi-level multiple imputation. This study is registered with PROSPERO (CRD42021272679).

**Findings:**

We obtained IPD from 16 national surveys out of 21 national and five sub-national surveys identified (five in Asia and 11 in Africa, N = 740,815). Across surveys, 15.1%–56.7% of TB were subclinical (median: 38.1%). In the multivariable model, current smoking was associated with both subclinical (OR 1.67, 95% CI 1.27–2.40) and symptomatic TB (OR 1.49, 95% CI 1.34–1.66). Self-reported diabetes was associated with symptomatic TB (OR 1.67, 95% CI 1.17–2.40) but not with subclinical TB (OR 0.92, 95% CI 0.55–1.55). For alcohol drinking ≥ twice per week vs no alcohol drinking, the estimates were imprecise (OR 1.59, 95% CI 0.70–3.62) for subclinical TB and OR 1.43, 95% CI 0.59–3.46 for symptomatic TB). For the association between current smoking and symptomatic TB, I^2^ was high (76.5% (95% CI 62.0–85.4), while the direction of the point estimates was consistent except for three surveys with wide CIs.

**Interpretation:**

Our findings suggest that current smokers are more likely to have both symptomatic and subclinical TB. These individuals can, therefore, be prioritised for intensified screening, such as the use of chest X-ray in the context of community-based screening. People with self-reported diabetes are also more likely to have symptomatic TB, but the association is unclear for subclinical TB.

**Funding:**

None.

**Research in context**

**Evidence before this study**

Low-income and middle-income countries are facing dual epidemics of tuberculosis (TB) and non-communicable diseases (NCDs). The World Health Organization (WHO) recommends systematic screening for TB in specific populations who are at an increased risk of TB to find people with TB who are not diagnosed and treated. Certain NCDs are known to increase the risk of TB. Previous systematic reviews have reported a 1.5–3.5-fold risk for developing TB in people with diabetes and around 2.5-fold risk for TB disease or TB infection in people who smoke. However, limited data exist on the risk of prevalent TB associated with these factors in the context of population-wide systematic screening in countries with a high TB burden. We aimed to conduct an individual participant data meta-analysis of national and sub-national TB prevalence surveys to synthesise the evidence on the risk of symptomatic and subclinical TB in people with NCD and/or NCD risk factors.

**Added value of this study**

Our meta-analysis included a large sample size (n = 740,815) from 16 nationally representative TB prevalence surveys conducted in Asian and African countries with a high level of TB incidence. The use of individual participant data enabled us to standardise the definition of subclinical TB and perform mixed-effects regression analyses to estimate the risk for different manifestations of TB. Our findings show, in models adjusted for age and gender, current smoking was associated with both subclinical (OR 1.62, 95% CI 1.38–1.90) and symptomatic TB (OR 1.49, 95% CI 1.34–1.66); self-reported diabetes was associated with symptomatic TB (OR 1.67, 95% CI 1.17–2.40), but not with subclinical TB (OR 0.92, 95% CI 0.55–1.55). The strength of these associations was smaller than those for past history of TB and HIV.

**Implications of all the available evidence**

Our findings could provide valuable data for decision makers when prioritising screening. For example, people who have self-reported diabetes and current smokers are more likely to have symptomatic TB, and thus they can be prioritised for systematic screening alongside other known risk groups, including people with HIV and those with a past history of TB. Conversely, people who smoke are more likely to have subclinical TB, in addition to symptomatic TB, than non-smokers are; therefore, current smokers might warrant intensified screening, such as the use of chest X-ray. It is worth noting that, in our analysis, not all surveys collected NCD-related variables systematically, precluding the development of a multivariable prediction model. Further surveys should consider collecting NCD-related variables systematically to aid the development of a model to predict individual TB risk associated with NCDs and other factors.

## Introduction

Annually over 10 million people develop tuberculosis (TB), and 1.6 million die from it globally.^1^ Under-detection of disease has been an obstacle, further compounded by the pandemic of COVID-19.^1^ The pandemic has disrupted essential health services, including those for TB. TB incidence increased from 10.1 to 10.6 million between 2020 and 2021 whereas the number of TB cases notified declined significantly to only 6.4 million compared to 7.1 million before the pandemic in 2019. Moreover, the number of TB deaths increased (from 1.2 million to 1.6 million) during the same period.^1^ One of the strategies that can help find more people with TB and place them on treatment is systematic screening. The World Health Organization (WHO) recommends systematic screening for active TB disease in specific populations or settings.^2^ Systematic screening intends to identify individuals who have TB disease, either symptomatic or asymptomatic, at the time of screening (i.e. prevalent TB).^2^ The target populations include, for example, the general population in areas with an estimated TB prevalence of 0.5% or higher and people living with human immunodeficiency virus (HIV).^2^ WHO also recommends systematic screening in people attending health facilities with clinical risk factors, such as diabetes and smoking, among others.^2^ WHO recommends prioritising groups for screening “based on their risk of TB, the risk of poor treatment outcomes if diagnosis is delayed and the size of the risk group in a given setting.”^2^ Quantifying the risk of prevalent TB in people with different factors allows countries to estimate the yield of systematic screening and help plan the targeted implementation of screening activities.^3^ Non-communicable diseases (NCDs), such as diabetes and NCD risk factors (e.g., smoking and alcohol use), are known to increase the risk for TB. For example, systematic reviews reported a 1.5–3.5-fold risk for developing TB in people with diabetes^4^^,^^5^ and around 2.5-fold risk for TB disease or TB infection in people who smoke.^6^ However, most studies underpinning the recommendations are based on case–control studies, cohort studies assessing incident TB, or studies using TB diagnosed through routine care.^2^^,^^4^^,^^5^^,^^7^ Limited data exist on the risk of prevalent TB associated with these factors in the context of population-wide systematic screening from countries with a high TB burden.

National TB prevalence surveys are population-based multi-stage cluster sampling surveys whose primary aim is to estimate the national prevalence of TB. Some prevalence surveys collected data on NCDs and NCD risk factors such as smoking, alcohol use, and self-reported diabetes. Using individual participant data (IPD) from these surveys enables quantifying the risk of prevalent TB by NCDs and NCD risk factors. However, no such IPD meta-analysis of prevalence surveys has been done to date.

A recent meta-analysis of aggregated data from TB prevalence surveys found that 36–80% of people with TB do not have symptoms yet bacteriologically positive, so-called subclinical TB; this highlights challenges in finding TB through symptom-based screening.^8^ While this meta-analysis was an essential first step to quantifying the burden of subclinical TB, the use of aggregated data precluded analysis to understand whether demographic and clinical factors can be used to predict the presence of subclinical TB. Such potential factors include NCDs and NCD risk factors (e.g., smoking), but the risk of different manifestations of TB associated with these factors is unknown. Understanding predictors could help prioritise X-ray-based screening for those who are more likely to have subclinical TB. In addition, in the previous review, the same definition of subclinical TB across surveys could not be applied because of the use of aggregated data and the definition of symptomatic TB varied by survey report or was not defined.^8^

Therefore, we conducted an IPD meta-analysis of national TB prevalence surveys. First, we aimed to quantify the proportion of subclinical TB using the standardised definition. Second, we investigated the risk of symptomatic and subclinical TB in people with NCDs and NCD risk factors compared to those without such factors in the context of population-level systematic screening.

## Methods

### Study design and ethics

This is a systematic review and IPD meta-analysis of TB prevalence surveys following the Preferred Reporting Items for Systematic Review and Meta-Analyses of individual participant data: the PRISMA-IPD Statement.^9^ The protocol of this systematic review has been pre-registered (https://www.crd.york.ac.uk/prospero/display_record.php?RecordID=272679).

This IPD meta-analysis was approved by the University College London Research Ethics Committee (18,969/001). All participants provided informed consent to participate in the primary surveys included in this meta-analysis.

### Search strategy and eligibility criteria

We included national and sub-national TB prevalence surveys in low and middle-income countries that reported at least one NCD or NCD risk factor (e.g., smoking, alcohol use) among participants.

Most national TB prevalence surveys follow a similar WHO-recommended standard protocol.^10^ Individuals aged 15 years or older identified through multi-stage random sampling undergo symptom screening and chest X-ray. Sputum samples are collected from participants who have symptoms or chest X-ray findings suggestive of TB (or any lung abnormality, depending on each survey).

We included surveys that collected at least one of the followings: diabetes, hypertension, chronic kidney disease, cardiovascular disease, chronic respiratory disease, smoking, harmful use of alcohol, and malnutrition (based on body mass index (BMI) or as defined by surveys). The diagnosis of NCDs followed the definitions used by surveys.

For the diagnosis of TB disease, we used survey cases as defined in each survey,^10^ which were confirmed bacteriologically either by culture or Gene Xpert. Subclinical TB was defined as bacteriologically-confirmed TB without any of the following symptoms of any duration: current cough, weight loss, fever, or night sweats.^11^ Symptomatic TB was defined as the presence of at least one of the above symptoms of any duration.

We identified eligible prevalence surveys through the list of national surveys maintained by WHO.^1^ Survey reports and protocols were used to identify eligible surveys. Additionally, we searched Medline (OVID) and Embase on 10 August 2021 to identify sub-national surveys published since 1 January 2000. The search was updated on 23 March 2023 to identify new surveys. The detailed search strategy is presented in Appendix 1.

Two investigators (YH and FM) independently reviewed titles and abstracts to identify potentially eligible studies in duplicate. The same two investigators reviewed full-text articles of those identified through the first screening. Discrepancies were resolved through discussion.

### Data collection and quality assessment

National TB programmes or equivalents or authors of the eligible surveys were invited to participate and share IPD (See Appendix 2 for the list of variables). We sought IPD from surveys found until 10 August 2021 (i.e. the initial search) to allow sufficient time for data cleaning and harmonisation, and analysis. For surveys found through the updated search, we sought aggregated data from published reports and by contacting the authors.

We checked data against the survey reports and resolved queries by contacting the original investigators. The frequency of alcohol drinking was classified somewhat differently by each survey (Table S1, Appendix 3). Considering those definitions, alcohol drinking was pragmatically classified into three groups: drinking ≥ twice per week, once a week or less, vs no drinking. Smoking history was classified into current smoking, past smoking, and never smoking.

Prevalence surveys were conducted in accordance with the methodology recommended by WHO, ensuring the representatives of the participants through random sampling and using recommended screening and diagnostic methods.^10^ Further, there is no well-established tool to assess the quality of cross-sectional studies focusing on assessing the association between exposures and outcomes. Thus, we conducted a quality assessment focusing on domains relevant to our analysis, incorporating those included in the Risk Of Bias In Non-randomised Studies–of Exposures (ROBINS-E).^12^ Accordingly, we assessed the participation rate to assess the risk of selection bias and methods for diagnosis of TB and NCDs to assess the risk of bias due to misclassification of exposure. Additionally, we collected information about TB screening methods and confirmatory diagnostics to assess the risk of bias due to misclassification of outcome and checked the missingness of exposure and outcome variables.

### Statistical analysis

#### Handling of missing data

We conducted multiple imputation using multi-level fully conditional specifications (see Appendix 3 for details). We generated 20 multiply imputed data sets with 20 iterations between successive imputation. All primary analyses were performed across multiply imputed datasets.

#### Descriptive analysis and regression models

We calculated the crude prevalence of TB and the proportion of subclinical TB by country using the imputed datasets. Clinical and demographic variables were presented by TB status.

We performed multi-level logistic regressions to estimate the odds ratio for all TB combining symptomatic and subclinical TB as well as multi-level multinomial regressions to estimate the odds ratio for subclinical and symptomatic TB, respectively, compared to people without TB (i.e. one-stage meta-analysis). Predictors of interest included current smoking, past smoking, alcohol use, diabetes, age, and gender. None of the included surveys determined prevalent diabetes with blood tests; they relied on self-reported diabetes instead. Self-reported diabetes has a low sensitivity of around 50% but a high specificity of over 95% against prevalent diabetes, including known diabetes and that newly diagnosed through laboratory tests.^13^^,^^14^ Despite this challenge, we included self-reported diabetes in our analysis to investigate whether this simple information could help identify individuals at higher risk for TB. This is particularly relevant in resource-limited settings where laboratory tests may be difficult to access. We did not include other NCDs due to unavailability of data. We also included HIV and past history of TB in order to compare the level of risk associated with smoking, alcohol use, and diabetes with risk factors that are recommended for systematic screening.^2^ We did not include socioeconomic status due to limited availability or ethnicity due to little within-survey variations. Smoking history was included in the models in two ways: 1) current smokers vs non-current smokers (including both never and past smokers) as a binary variable and 2) current smokers, past smokers vs never smokers, as a categorical variable. The binary variable was intended to assess if simply checking for current smoking can be used to identify people who are likely to have all TB as well as specific manifestations of TB. The categorical variable was to assess if past smoking is still associated with TB risk as opposed to never-smoking. We conducted univariable and multivariable modelling. In the univariable model, we included each of the above risk factors one at a time. Subsequently, we conducted multivariable modelling, adding current smoking, past smoking, alcohol use, diabetes, past history of TB, and HIV one at a time, adjusted for age and gender alone, to examine if they can be used to identify individuals who are more likely to have TB overall or TB with specific manifestations hence can be prioritised for systematic screening, regardless of age and gender. The present analysis was not intended to examine causal associations. The models included random intercepts for surveys and households to account for clustering.

We explored the heterogeneity in the adjusted odds ratios between countries through forest plots. We quantified the proportion of total variability due to between-study heterogeneity by calculating I-squared (by fitting a two-stage model).

As a sub-group analysis, we repeated the above analysis in HIV-negative individuals, given that people living with HIV are already prioritised for systematic screening regardless of the presence of other risk factors.^15^ This was done by excluding HIV-positive participants and participants with unknown HIV status before multiple imputation.

#### Sensitivity analysis

First, we explored different categorisations of alcohol drinking: 1) any drinking vs no drinking; and 2) drinking ≥ twice per week vs drinking < twice per week.

Second, we repeated the analyses by excluding: 1) Tanzania alone due to a concern about the validity of the number of bacteriologically positive cases,^16^ 2) countries that collected NCD data only among a subset of the participants, and 3) countries that did not collect all four symptoms.

Third, for alcohol drinking and diabetes, because of the systematically missing data and a large proportion of sporadically missing data in some surveys, we repeated the analyses by restricting to studies with minimal missing data.

Fourth, to explore the impact of misclassification of self-reported diabetic status, we conducted a record-level sensitivity analysis assuming different levels of sensitivity and specificity of self-reported diabetes (See Appendix 3 for details).

We did not anticipate the publication bias since the WHO maintains a list of national TB prevalence surveys, and hence it was not examined.

### Role of the funding source

There was no funding source for this study.

## Results

### Characteristics of included studies

**Fig. 1:**
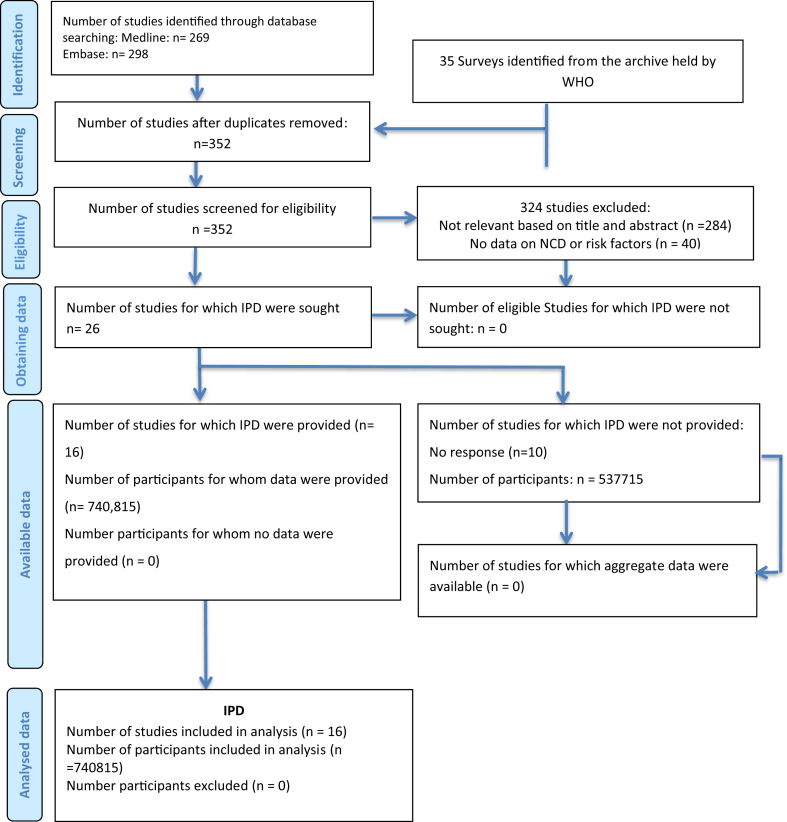
**Study selection-results of the initial search.** IPD: individual participant data; WHO: World Health Organization; NCDs: non-communicable diseases.

From the archive of TB prevalence surveys held by WHO, 21 surveys were found eligible (Fig. 1). Sixteen (73%) agreed to share datasets and were included in the meta-analysis (740,815 participants in total).^17^, ^18^, ^19^, ^20^, ^21^, ^22^, ^23^, ^24^, ^25^, ^26^, ^27^, ^28^, ^29^, ^30^, ^31^, ^32^ Five surveys (in Democratic People's Republic of Korea, Ethiopia, Myanmar, Rwanda, and Zimbabwe) did not respond to our request. All of them had data on smoking, two on diabetes, and two on alcohol use. When stratified by the availability of NCDs-related variables, for smoking, diabetes, and alcohol use, we included 16 out of 22 eligible national surveys, nine out of 11, and eight out of 10, respectively. The database searches identified an additional five eligible studies from Ethiopia,^33^ India,^34^ Viet Nam,^35^ and South Africa and Zambia^36^; however, none of the studies responded to our request before the closure of data collection. All of them were subnational surveys comprising a total of 286,340 participants; each collected data on smoking only as NCD risk factor. The updated search found one national survey in India^37^ and one sub-national survey in India^38^ that reported the association of NCDs and NCD-related risk factors with TB (Fig. S1).

Our meta-analysis included 16 national TB prevalence surveys conducted between 2012 and 2020, including 5 in Asia and 11 in Africa (Table S2). All surveys included individuals aged 15 years or older. Thirteen studies used sputum smear and culture with or without Xpert MTB/RIF to diagnose TB among participants with TB-suggestive symptoms and/or chest X-ray findings, while the rest^23^^,^^26^^,^^30^ used culture and Xpert MTB/RIF or Xpert MTB/RIF Ultra without smear (Table S3). In all surveys, there were fewer male participants than females; the proportion of male participants ranged from 39.6 to 46.6%. Of TB cases diagnosed, 3.0% to 11.4% were on treatment. We did not find issues that could undermine IPD integrity.

Nine surveys collected data on self-reported diabetes.^17^, ^18^, ^19^, ^20^, ^21^, ^22^, ^23^^,^^30^^,^^32^ All studies had data on current smoking vs non-current smoking, and 13 additionally had data on past smoking. Eight had information on alcohol use.^18^^,^^20^, ^21^, ^22^, ^23^^,^^28^^,^^30^ Only four surveys collected data on BMI.^18^^,^^21^^,^^22^^,^^24^ No surveys reported data on other types of NCDs such as chronic kidney disease. In six surveys, data on NCDs and/or their risk factors were collected in a subset of participants: participants eligible for sputum collection and a randomly selected subset of other participants in Eswatini, Namibia, and Mozambique,^21^^,^^23^^,^^31^ those eligible for sputum collection in the United Republic of Tanzania, and Viet Nam,^22^^,^^32^ and participants who had cough ≥ two weeks, had TB diagnosis, or treatment history in Ghana.^20^ As a result, these six surveys had a large proportion of missing data, for example, 75.3–95.7% for diabetes (Table S2 and Figure S2). HIV status was collected in nine surveys, including three in which HIV status was sought only in a subset of the participants.^22^^,^^23^^,^^29^ In the remaining six surveys, HIV status was missing in <0.01%–29.6%.

Five surveys used cough ≥ two weeks alone,^18^^,^^20^^,^^24^^,^^29^^,^^32^ and two^17^^,^^19^ used cough ≥ two weeks or blood in sputum/haemoptysis as symptom screening criteria to select participants for sputum testing. The remaining countries included additional symptoms such as fever, weight loss, and night sweats (Table S3).

Three surveys did not collect all of the four TB symptoms (current cough, fever, night sweats, and weight loss) required to define subclinical TB from all participants.^20^^,^^22^^,^^24^ Current cough was not collected in the survey in the United Republic of Tanzania, and night sweats were not in Nigeria. The survey in Ghana asked about fever, weight loss, and night sweats only in 2819 individuals who had cough > two weeks, prevalent TB, or TB treatment history. Additionally, Viet Nam collected fever, weight loss and night sweats only in participants who were eligible for sputum submission. The number of participants with or without any of the four symptoms was imputed through multi-level multiple imputation. The subsequent analyses were based on multiply imputed datasets.

Two surveys found through the updated search reported data on smoking, diabetes, and alcohol use, and its association with bacteriologically confirmed TB, regardless of symptoms.^37^^,^^38^ They did not provide data that could be included in the meta-analysis. Their characteristics and findings are summarised in Table S4.

### Characteristics of subclinical TB and symptomatic TB

The crude TB prevalence, not accounting for cluster sampling design, ranged from 0.28% in Bangladesh to 1.07% in the Philippines (Table S5). Among TB cases, 15.1% (Indonesia) to 56.7% (South Africa) met the definition of subclinical TB (median: 38.1%; interquartile range: 25.5–48.2%). Table 1 presents the characteristics of participants stratified by TB status: people without TB, those with subclinical TB, and those with symptomatic TB. The mean age was higher in people with subclinical TB (48.2 years) and symptomatic TB (45.9 years) than in those without TB (38.0 years). People meeting either TB case definition tended to be male, current smokers, HIV-positive, and had a past history of TB than those without TB. Diabetes was most common in people with symptomatic TB (6.4%), and it was more common in people with subclinical TB (4.1%) than those without TB (2.8%).

**Table 1:** Characteristics of participants by TB status.

Variable	Without TB	Subclinical TB	Symptomatic TB
Age, mean (SD)	38 (17.3)	48.2 (18.6)	45.9 (18.1)
Male, n (%)	312,154 (42.3)	729 (60.9)	1440 (64.1)
Female, n (%)	425,218 (57.7)	469 (39.1)	805 (35.9)
Current smoker, n (%)	144,491 (19.6)	430 (35.9)	798 (35.5)
Past smoker, n (%)	43,929 (6.0)	142 (11.8)	370 (16.5)
Alcohol drinking once a week or less, n (%)	166,737 (22.6)	346 (28.9)	590 (26.3)
Alcohol drinking twice a week or more, n (%)	42,903 (5.8)	117 (9.8)	213 (9.5)
Diabetes, n (%)^a^	20,401 (2.8)	49 (4.1)	145 (6.4)
HIV-positive, n (%)	78,569 (10.7)	207 (17.3)	427 (19.0)
Past history of TB, n (%)	24,024 (3.3)	132 (11.0)	371 (16.5)

Note: Based on multiply imputed datasets.

TB: tuberculosis; SD: standard deviation.

a Self-reported.

### Associations between NCDs, NCD risk factors, and TB status

In the univariable model, older age, male gender, history of TB, current smoking, and HIV status were all associated with a higher likelihood of all TB combined. This was true when assessed separately for symptomatic and subclinical TB (Table 2). For example, the male gender was associated with a 2-fold higher risk of prevalent TB (OR 2.14; 95% CI 1.89–2.42 for subclinical TB, and OR 2.46; 95% CI 2.25–2.69 for symptomatic TB). Similarly, current smoking was associated with a 2-fold higher risk (OR 2.24; 95% CI 1.94–2.60 for subclinical TB, and OR 2.21; 2.00–2.44 for symptomatic TB) compared to non-current smokers. Additionally, past smokers were more likely to have both subclinical and symptomatic TB (OR 2.54; 95% CI 1.72–3.74 for subclinical TB, and OR 3.87; 2.73–5.47 for symptomatic TB) than never smokers. In contrast, diabetes was associated with a 2-fold higher risk of symptomatic TB (OR 2.30; 95% CI 1.63–3.25) but not significantly with subclinical TB (OR 1.42; 95% CI 0.85–2.35). For alcohol drinking, the point estimates showed a higher likelihood of both subclinical and symptomatic TB, but the confidence intervals were wide, overlapping one.

**Table 2:** Associations between NCDs, NCD risk factors, and different manifestations of TB- multinomial logistic regression.

	All TB		Subclinical TB		Symptomatic TB	
	Odds ratio (95% CI)	p-value	Odds ratio (95% CI)	p-value	Odds ratio (95% CI)	p-value
Current smoker vs non-current smoker	2.22 (2.04–2.43)	<0.0001	2.24 (1.94–2.6)	<0.0001	2.21 (2.00–2.44)	<0.0001
Past smoker vs never smoker	3.38 (2.41–4.73)	<0.0001	2.54 (1.72–3.74)	<0.0001	3.87 (2.73–5.47)	<0.0001
Alcohol drinking once a week or less vs no alcohol drinking	1.28 (0.95–1.73)	0.098	1.4 (1.02–1.94)	0.041	1.22 (0.88–1.68)	0.22
Alcohol drinking ≥ twice per week vs no alcohol drinking	1.77 (0.78–4.02)	0.16	1.86 (0.84–4.13)	0.12	1.72 (0.73–4.03)	0.2
Diabetes	1.99 (1.42–2.78)	0.00021	1.42 (0.85–2.35)	0.17	2.3 (1.63–3.25)	<0.0001
Past history of TB	4.6 (4.14–5.12)	<0.0001	3.33 (2.74–4.05)	<0.0001	5.34 (4.72–6.03)	<0.0001
HIV	2.31 (1.56–3.42)	0.0002	2.17 (1.41–3.33)	0.001	2.39 (1.58–3.61)	0.00022
Age per 10-year increase	1.29 (1.27–1.31)	<0.0001	1.34 (1.3–1.38)	<0.0001	1.26 (1.23–1.29)	<0.0001
Male gender	2.34 (2.18–2.52)	<0.0001	2.14 (1.89–2.42)	<0.0001	2.46 (2.25–2.69)	<0.0001

NCDs: non-communicable diseases; TB: tuberculosis; CI: confidence interval; HIV: human immunodeficiency virus.

When the model included age and gender, the magnitude of the risk was highest in people with a past history of TB for all TB (OR 3.56; 95% CI 3.19–3.97) and for both symptomatic (OR 4.19; 95% CI 3.70–4.75) and subclinical TB (OR 2.51; 95% CI 2.06–3.06). This was followed by positive HIV status (Table 3). Current smoking was associated with both subclinical (OR 1.62, 95% CI 1.38–1.90) and symptomatic TB (OR 1.49; 95% CI 1.34–1.66). Past smoking was not significantly associated with subclinical TB (OR 1.41; 95% CI 0.91–2.18) in contrast to the significant association with symptomatic TB (OR 2.32: 95% CI 1.54–3.48). Similar to observations in the univariable model, diabetes was associated with symptomatic TB (OR 1.67, 95% CI 1.17–2.40), but not with subclinical TB (OR 0.92; 95% CI 0.55–1.55). In the model adjusted for age and gender, the point estimates for alcohol drinking ≥ twice per week vs no alcohol drinking were consistent with an increased risk for both, but the confidence intervals were wide, overlapping the null.

**Table 3:** Associations between NCDs, NCD risk factors, and different manifestations of TB, adjusted for age and gender.

	All TB		Subclinical TB		Symptomatic TB	
	Odds ratio (95% CI)	p-value	Odds ratio (95% CI)	p-value	Odds ratio (95% CI)	p-value
Current smoker vs non-current smoker	1.53 (1.39–1.69)	<0.0001	1.62 (1.38–1.9)	<0.0001	1.49 (1.34–1.66)	<0.0001
Past smoker vs never smoker	1.97 (1.33–2.91)	0.0016	1.41 (0.91–2.18)	0.12	2.32 (1.54–3.48)	0.00028
Alcohol drinking once a week or less vs no alcohol drinking	1.2 (0.91–1.58)	0.18	1.33 (0.98–1.8)	0.065	1.14 (0.84–1.54)	0.38
Alcohol drinking ≥ twice per week vs no alcohol drinking	1.49 (0.64–3.48)	0.34	1.59 (0.7–3.62)	0.26	1.43 (0.59–3.46)	0.41
Diabetes	1.39 (0.98–1.97)	0.063	0.92 (0.55–1.55)	0.75	1.67 (1.17–2.4)	0.0064
Past history of TB	3.56 (3.19–3.97)	<0.0001	2.51 (2.06–3.06)	<0.0001	4.19 (3.7–4.75)	<0.0001
HIV	2.39 (1.6–3.57)	0.00017	2.21 (1.42–3.43)	0.001	2.5 (1.64–3.81)	0.00016

NCDs: non-communicable diseases; TB: tuberculosis; CI: confidence interval; HIV: human immunodeficiency virus.

**Fig. 2:**
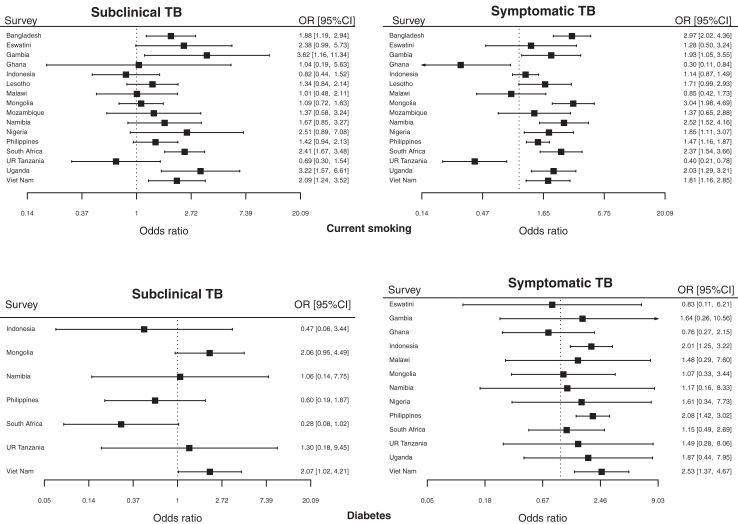
**The associations between current smoking/diabetes and TB status by survey**. Note: Results of multivariable multiple regression models adjusted for age and gender by survey. Surveys with large standard errors resulting in 95% confidence intervals ranging from 0 to infinity or for which the model failed to converge are excluded from the plots. TB: tuberculosis; OR: odds ratio; CI: confidence intervals. **Current smoking**: Subclinical TB: I-squared = 47.2% (95% CI 5.5–70.5), p = 0.019, tau^2^ = 0.08. Symptomatic TB: I-squared = 76.5% (95% CI 62–85.4), p < 0.0001, tau^2^ = 0.24. **Diabetes**: Subclinical TB: I-squared = 0% (95% CI 0–52.3), p = 0.74, tau^2^ = 0.37. Symptomatic TB: I-squared = 0% (95% CI 0–52.3), p = 0.8, tau^2^ = 0.011.

For the association between current smoking and subclinical TB, I^2^ was 47.2% (95% CI 5.5–70.5) (Fig. 2). I^2^ was larger for the association between current smoking and symptomatic TB (I^2^ = 76.5%; 95% CI 62.0–85.4). Nonetheless, the direction of the association was consistently above one except in three surveys (Ghana, Malawi, and the United Republic of Tanzania) with wide confidence intervals. For alcohol drinking, diabetes, and HIV, between-study heterogeneity accounted for little total variation (Fig. 2, Figure S3–S5). When restricted to HIV-negative participants, the associations between smoking and all TB and with subclinical and symptomatic TB remained similar (Table S6). The subgroup analysis resulted in the exclusion of surveys where HIV status was not collected, including Ghana, Indonesia, Philippines, and Viet Nam, which collected diabetes. The subgroup analysis did not show significant associations between diabetes and all TB and with subclinical and symptomatic TB.

### Sensitivity analysis

When using different categorisations of alcohol drinking, estimates remained imprecise with wide confidence intervals overlapping one; thus, it was difficult to see a difference in the results compared to the primary categorisation (Table S7).

We compared estimated odds ratios in the primary analysis with those excluding the United Republic of Tanzania, those excluding six surveys that collected NCD data only in a subset of participants, and those excluding three surveys that did not collect all four symptoms (Figure S7–S9). For current smoking, the exclusion of the six countries increased point estimates marginally (OR 1.49; 95% CI 1.34–1.66 in the primary analysis for symptomatic TB VS. OR 1.75; 95% CI 1.54–1.98). Overall, the odds ratios for past TB was reduced when excluding the six countries (e.g., OR 4.19; 95% CI 3.70–4.75 in the primary analysis for symptomatic TB VS. OR 3.35; 95% CI 2.85–3.95). The exclusion of the six countries resulted in a higher odds ratio for the association between alcohol drinking ≥ twice per week and subclinical TB (OR 2.14; 95% CI 1.16–3.94). This was similar when the analysis was restricted to three studies with minimal missing data on alcohol drinking (Table S8). Otherwise, the estimates were not substantially different in the sensitivity analyses.

Figure S10 presents the sensitivity analysis exploring the impact of the misclassification of diabetic status. In general, as the sensitivity of self-reported diabetes in people with TB increases (i.e. diabetes is more likely to be diagnosed in people with TB), ORs tend to become lower, suggesting that in the presence of underdiagnosis of diabetes, the estimated ORs are underestimated. For symptomatic TB, the lower limit of the uncertainty intervals crossed one only when there was a large difference in sensitivity (40% for people without TB and ≥70% for people with TB), and the specificity was 99%. For subclinical TB, the true association with diabetes tended to be positive in most scenarios in contrast to the association using the original diabetic status, especially if the specificity was 98%, suggesting the underestimation of the true association.

## Discussion

Our IPD meta-analysis reports data to suggest that, in addition to a history of TB and HIV, self-reported diabetes and current smoking could be used to identify people who are more likely to have prevalent TB, independent of age and gender. The magnitude of the risk was around 1.5-fold for both current smoking and self-reported diabetes (for symptomatic TB), and these estimates can guide screening policy and planning. Although the magnitude of the risk was higher for HIV and past history of TB, the prevalence of diabetes and current smoking is larger than or similar to that of HIV or past history of TB in some countries. For example, in the Philippines, the prevalence of HIV is <1%, whereas the prevalence of current smoking among adults is 6.5% for females and 39% for males, and the prevalence of diabetes is 7%.^1^ In such a case, systematically targeting individuals with those risk factors can help find more people with TB. While screening TB among people with diabetes has been recommended for over a decade,^39^ only 15 of 30 high TB burden countries recommended it in their guidelines and data on the level of implementation is lacking.^40^ Current smoking was associated with both symptomatic and subclinical TB. This suggests current smokers could be prioritised for chest X-ray in addition to symptom screening.

Interestingly, self-reported diabetes was associated with an increased likelihood of symptomatic TB but not with subclinical TB. The increased risk is consistent with previous studies that reported a 1.5-3 fold increase in the risk for TB.^4^^,^^5^ Most of which were cohort and case–control studies. Case-control studies usually use TB diagnosed through routine care as cases, so it is likely that TB in those studies tended to be symptomatic. A review by Al-Rifai included three cross-sectional studies that estimated the association between TB and NCDs.^4^^,^^41^, ^42^, ^43^ None of them applied systematic screening for TB, instead using past history of TB, TB-suggested symptoms, or TB diagnosed through routine care. Thus, the observed associations in previous studies were likely to be more applicable to symptomatic TB. In fact, studies suggested that TB tends to be more severe and more likely to be symptomatic in people with diabetes than those without.^44^^,^^45^ In contrast, there is limited data on the risk of subclinical TB in people with diabetes. If the risk of subclinical TB is not increased in people with diabetes as suggested by the results of our primary analysis, chest X-ray in asymptomatic people with diabetes might not lead to finding more than what is expected from the background TB prevalence. However, this does not rule out the use of X-rays, taking into account the expected yields and resource availability. Furthermore, it should be noted that the confidence intervals of the pooled and individual estimates by country are large. In addition, as shown in the sensitivity analysis, the association were likely to be underestimated, given the use of self-reported diabetes. Thus, the increase in the risk of subclinical TB is not yet ruled out, but the magnitude of the risk might be lower than that for symptomatic TB.

The magnitude of the TB risk associated with current smoking varied significantly across surveys. This could be explained by different social contexts, such as how smoking is associated with more time spent in settings with a higher risk of TB exposure (e.g., bars). Since our scope was not causal inference, confounding variables such as lifestyle might have caused the observed association. Regardless of the mechanism behind the association, the increased risk suggests that higher yields of subclinical and symptomatic TB are expected in current smokers compared to the general population; therefore, targeted systematic screening may help find people missed to be diagnosed with TB. However, given the heterogeneity, countries should consider their local data and contexts instead of universally applying pooled estimates. Two (Ghana and the United Republic of Tanzania) countries had inverse associations that were statistically significant. This might be explained by bias due to the collection of smoking history only in selected participants (e.g., eligible for sputum submission), and in Ghana, other symptoms than cough were collected only in participants who had cough ≥ two weeks, TB or TB treatment history. Other possible reasons might be the cessation of smoking in symptomatic individuals and chance finding (given the wide confidence intervals and multiple analyses).

In the primary analysis, alcohol drinking was surprisingly not significantly associated with TB. In contrast, it was significant in the sensitivity analysis that excluded studies with a large proportion of missing data though the magnitude of the risk was not markedly different. This suggests that the finding is not robust because of the amount of missing data, and the association remains inconclusive.

While it was not our primary scope of the review, we confirmed prior knowledge that males are more likely to have TB, yet, they were underrepresented in all surveys. This suggests that in community-based screening activities, male people may be less likely to present, which would reduce screening yields. Thus, screening activities should ensure that they are engaged to maximise the yield and cost-effectiveness of screening activities.

The strength of the present study is the large sample size (over 700,000), combining data from nationally representative surveys both in Asian and African countries with a high level of TB incidence. The availability of IPD enabled the standardisation of the definition of subclinical TB. There are several limitations. First, the diagnosis of diabetes was based on self-report. Thus, under-detection is likely given the low sensitivity of self-reported diabetes, albeit with high specificity.^13^^,^^14^ We showed through the sensitivity analysis that the association between diabetes and symptomatic TB was robust. For instance, in a Demographic and Health Survey in South Africa, 13 and 8% of men and women, respectively, had diabetes based on HbA1c measurements, in contrast to 5% and 4% based on self-report, suggesting the underdiagnosis of diabetes.^46^ Nevertheless, the strength of the risk found in the review might not be generalised to settings where diabetes is identified through systematic screening. On the other hand, laboratory-based screening for diabetes might not be available or feasible in a community-based screening set-up. Asking about self-reported diabetes could be a simple tool to identify people more likely to have TB, especially in settings with a high prevalence of diabetes. In such a case, the magnitude of the risk estimated in our study based on self-reported diabetes could be applied to gauge the expected increase in screening yields.

Second, alcohol use and diabetes were not collected in all surveys, and to account for this, data were imputed through multi-level multiple imputation. In six surveys, diabetes, alcohol, and smoking were collected only in a subset of the participants. Still, in three of them, the information was collected in all participants who were eligible for sputum submission as well as those who did not and were randomly selected. Hence, the imputation model including eligibility for sputum submission was likely to impute missing data without bias under a reasonable missingness mechanism (missing at random conditional on all observed variables). Furthermore, the sensitivity analysis did not show significant differences in the findings. Similarly, HIV status was not collected in all surveys, and even when collected, it was sporadically missing partly due to refusal. However, the impact of non-response bias in national surveys was not reported to be large,^47^ and the use of multiple imputation can incorporate the uncertainty.^48^ Third, three surveys (Ghana, Nigeria, and the United Republic of Tanzania) did not collect all four TB suggestive symptoms used to define symptomatic TB. While the presence of at least one of the symptoms that were collected is sufficient to deem one as symptomatic, when they are absent, missingness of the other symptoms results in missing symptomatic status. Hence, missingness was not at random. The use of information from other surveys through multi-level modelling helped recover information, but there is a possibility of bias. It is, however, reassuring that the findings were mostly consistent in the sensitivity analyses. Fourth, multi-level multiple imputation did not account for clustering within households; thus, the imputation model was not fully compatible with the analysis model where random household effects were incorporated, which could have led to small biases, particularly in the estimation of standard errors. However, to our knowledge, no available software could allow for this while retaining all the flexibility of our approach, and hence our method is likely to be the best among practical alternatives in minimising bias. Fifth, all surveys collected sputum only when participants met screening criteria comprising X-ray findings and symptoms. While this is a standard methodology for TB prevalence surveys recommended by WHO,^10^ subclinical TB without apparent lung shadows might have been missed. There were differences in the symptom screening criteria; however, all but one study used any chest X-ray lung abnormality as another criterion, which has a high sensitivity of around 95%.^37^^,^^49^ Sixth, we did not include IPD from two studies identified in our updated search due to the sufficient time required to retrieve and incorporate them into our analysis. Despite differences in covariates that preclude a direct comparison of our multivariable model results with theirs, their univariable model results align with ours, pointing to an elevated TB risk among current smokers and individuals with diabetes in both studies. The inclusion of these studies likely doesn't alter the overall interpretation of our findings. Lastly, we did not explore the risk of TB due to the presence of overlapping risk factors. A large proportion of missing data in some variables and the relatively small number of TB cases identified precluded performing more advanced modelling, such as the inclusion of interaction terms. Standardisation of the collection of NCD-related variables in accordance with the WHO STEPwise approach^50^ in future TB prevalence surveys could address this gap and help develop a model to estimate individual risk for TB, including subclinical TB, through multivariable modelling. The collection of local data is even more important in light of heterogeneity in the risk shown in our analysis.

The present study suggests that people who have self-reported diabetes and current smokers are more likely to have symptomatic TB. Up to 50% of TB can be subclinical, and people who smoke are more likely to have subclinical TB, independent of age and gender. Current smokers might warrant intensified screening, such as the use of chest X-ray, taking into account the expected yields. Future surveys should consider the collection of NCD-related variables systematically to enable more granular analysis and develop a model to predict individual TB risk associated with NCDs.

## Contributors

YH, MQ, IA, AC, and MXR designed the study protocol. YH and FM did the systematic review. YH analysed the data with assistance and advice from IL, MQ, IA, AC, and MXR. The following authors contributed individual data, data curation, editing, and advisement on the manuscript results: FAB, IMOA, YAP, UDA, AOB, VB, DBL, TB, TD, SD, BD, SE, MNF, KF, AMCGG, DMGG, MMH, FI, MK, DVK, SK, KK, BK, EK, ZK, IL, AL, DM, IM, LBMM, SMf, SMo, JM, TM, DEM, LM, HBN, HVN, LP, PP, MahmR, MahbR, MSR, TR, PR, NR, ER, MFR, MS, ARS, WS, CS, TSo, TSt, SS, OS, AMT, KT, MVDW, SW, and MMZ. YH wrote the first draft of the manuscript, which was revised based on comments from co-authors. All authors approved the final version of the manuscript. YH and IL accessed and verified the underlying data, and YH had the final responsibility for the decision to submit it for publication.

## Data sharing statement

The IPD database is stored within the UCL Data Repository and can be shared subject to the approval of the corresponding authors of the original studies.

## Declaration of interests

TSo declares a receipt of funding from the Global Fund for conducting the TB prevalence survey in Mongolia. All other authors declare no competing interests.
