# Supplementary material for: Association of diabetes, smoking, and alcohol use with subclinical-to-symptomatic spectrum of tuberculosis in 16 countries: an individual participant data meta-analysis of national tuberculosis prevalence surveys
Source: eClinicalMedicine. 2023 Aug 30;63:102191. doi: 10.1016/j.eclinm.2023.102191 (PMC10480554; doi:10.1016/j.eclinm.2023.102191)
Supplement: Supplementary Material [file mmc1.docx]

Contents

[Appendix 1. Search strategy 2](#_Toc137567753)

[Appendix 2. Variables that were requested 3](#_Toc137567754)

[Appendix 3. Supplementary methods 4](#_Toc137567755)

[Appendix 4. Supplementary figures and tables 7](#_Toc137567756)

[**Table S1. Categorizations of current alcohol drinking by surveys** 7](#_Toc137567757)

[**Figure S1. Results of the updated literature search** 8](#_Toc137567758)

[**Table S2-1. Characteristics of included national TB prevalence surveys** 9](#_Toc137567759)

[**Table S2-2. Characteristics of included national TB prevalence surveys** 11](#_Toc137567760)

[**Table S3. Quality of individual surveys** 13](#_Toc137567761)

[**Figure S2.Proportion of missing data by variable and by survey** 15](#_Toc137567762)

[**Table S4. Studies found through the updated literature search** 16](#_Toc137567763)

[**Table S5. Crude prevalence of active TB and proportion of subclinical TB** 17](#_Toc137567764)

[**Figure S3. Past smoking and TB status by survey** 18](#_Toc137567765)

[**Figure S4. Alcohol drinking and TB status by survey** 19](#_Toc137567766)

[**Figure S5. HIV status and TB status by survey** 21](#_Toc137567767)

[**Figure S6. Past history of TB and TB status by survey** 22](#_Toc137567768)

[**Table S6. Associations between NCDs, NCD risk factors, and different manifestations of TB, adjusted for age and sex, in HIV-negative individuals** 23](#_Toc137567769)

[**Table S7. Sensitivity analysis using different categorisations of alcohol drinking** 24](#_Toc137567770)

[**Figure S7. Sensitivity analysis for the associations between all TB and predictors adjusted for age and gender** 25](#_Toc137567771)

[**Figure S8. Sensitivity analysis for the associations between subclinical TB and predictors adjusted for age and gender** 26](#_Toc137567772)

[**Figure S9. Sensitivity analysis for the associations between symptomatic TB and predictors adjusted for age and gender** 27](#_Toc137567773)

[**Table S8. Associations between NCDs, NCD risk factors, and different manifestations of TB, adjusted for age and sex, restricting to surveys with minimal missing data** 28](#_Toc137567774)

[**Figure S10. Sensitivity analysis-impact of misclassification of diabetic status** 29](#_Toc137567775)

# Appendix 1. Search strategy

Medline

| 1 | tuberculosis.m_titl. |
| --- | --- |
| 2 | prevalence.m_titl. |
| 3 | survey.tw. |
| 4 | 1 and 2 and 3 |
| 5 | limit 4 to yr="2000 -Current" |

EMBASE

| 1 | tuberculosis.m_titl. |
| --- | --- |
| 2 | prevalence.m_titl. |
| 3 | survey.ti,ab,kw. |
| 4 | 1 and 2 and 3 |
| 5 | limit 4 to yr="2000 -Current" |

# Appendix 2. Variables that were requested

| **Household level information** |
| --- |
| Cluster ID |
| Household ID |
| Availability of assets (e.g. refrigerator) |
| Access to clean water |
| Use of biomass fuel |
| Number of rooms |
| Household Income |
| Other variables relevant to socioeconomic status collected in surveys. |
| Number of household members |
| Education status |
| **Individual data** |
| Sex |
| Age |
| Household id |
| Smoking |
| Alcohol use |
| HIV status |
| Body weight |
| Body mass index |
| Occupation |
| Education level |
| Diabetes |
| Hypertension |
| Silicosis |
| Chronic obstructive pulmonary disease |
| Asthma |
| Past history of TB |
| Current TB treatment |
| Symptoms |
| Chest X-Ray abnormality |
| Smear microscopy result |
| Xpert MTB/RIF result |
| Sputum Culture result |

# Appendix 3. Supplementary methods

*Handling of missing data*

We conducted multiple imputation using multi-level fully conditional specifications, including the following variables in the imputation model: tuberculosis (TB) as a categorical variable with three groups (no TB, subclinical TB, and symptomatic TB), predictors (diabetes, alcohol use, smoking history, previous history of TB, age, gender), and auxiliary variables (TB symptoms and chest X-ray findings). All of these variables, except age and gender, had sporadically and/or systematically missing data, and they were imputed. This means data were imputed regardless of the collection of some non-communicable diseases (NCDs)-related variables or HIV status. We also included a binary indicator for African countries vs Asian countries in the model to ensure the imputation of human immunodeficiency virus (HIV) status results in plausible prevalence in the region. The model accounted for clustering within surveys. In addition, we intended to account for clustering within households and/or sampling clusters. However, the model failed to converge, most likely due to very low variation within households; therefore, we only accounted for clustering within the surveys.

We generated 20 multiply imputed data sets with 20 iterations between successive imputation. Model convergence was assessed visually by examining trace plots. All primary analyses were performed across multiply imputed datasets; substantive models were fitted on each imputed dataset, and their outputs were combined using Rubin's rules.^1^

*Sensitivity analysis- quantitative bias analysis*

To explore the impact of misclassification of self-reported diabetic status, we conducted a record-level sensitivity analysis assuming different levels of sensitivity and specificity of self-reported diabetes. We tested both non-differential and differential misclassification of diabetic status by the presence of TB. Based on previous studies, we assumed the sensitivity of self-reported diabetes to be 40% or 50% in people without TB.^2,3^ In people with TB, we tested the same levels of sensitivity as in those without TB (i.e. non-differential misclassification) as well as higher levels, ranging from 50% to 80%. This allowed us to examine if a higher likelihood of diabetes being diagnosed in people with TB than in those without TB can lead to spurious associations between self-reported diabetes and TB. The prevalence of self-reported diabetes in the study population was 2.8% in people without TB (see Results). This means the specificity is at least 97.2% in this population, which is consistent with the high specificity reported in the literature.^2,3^ Hence, we tested 98% and 99% specificity. We adapted the approach described by Fox et al. while using fixed levels of sensitivity and specificity.^4^ We first sampled one of the 20 multiply imputed datasets and estimated positive and negative predictive values for diabetes given the observed diabetic status. Second, using the predictive values, we simulated a new variable representing true diabetic status drawing at random from a Bernoulli distribution. We fitted a multinomial regression model using the new variable as an exposure adjusted for age and gender. Finally, to account for random errors, we sampled a standard normal deviate and multiplied it by the standard error of the bias-adjusted association and combined it with the point estimate from the model. We repeated the above process 1000 times and presented the median and 2.5^th^ and 97.5^th^ percentiles as uncertainty intervals. To reduce the computation time, the multinomial model excluded random intercepts for households. We compared the results with those from the models using the reported diabetic status.

**References**

1. Rubin, D. B. Multiple Imputation for Nonresponse In Surveys (Wiley-Interscience, 2004).

2. Ning M, Zhang Q, Yang M. Comparison of self-reported and biomedical data on hypertension and diabetes: findings from the China Health and Retirement Longitudinal Study (CHARLS). *BMJ Open* 2016; **6**(1): e009836.

3. Schneider AL, Pankow JS, Heiss G, Selvin E. Validity and reliability of self-reported diabetes in the Atherosclerosis Risk in Communities Study. *Am J Epidemiol* 2012; **176**(8): 738-43.

4. Fox MP, MacLehose RF, Lash TL. Probabilistic Bias Analysis for Simulation of Record-Level Data. Applying Quantitative Bias Analysis to Epidemiologic Data Second Edition. Switzerland: Springer; 2021: 291-326.

# Appendix 4. Supplementary figures and tables

## **Table S1. Categorizations of current alcohol drinking by surveys**

| Eswatini | None  Once a week  Monthly or less  2-4 times a month  2-3 times a week  4 or more times a week |
| --- | --- |
| Gambia | None  Occasionally 1-2 times/wk 3-5 times/wk > 5 times/wks |
| Ghana | None  Once in past year Once in 6 months Once in a month Once in a week 3-4 times a week Everyday |
| Mongolia | None Once a month or less 2-4 times a month 2-3 times a week At least 4 times a week |
| Mozambique | None  1 times a month or less  2 to 4 times a month  2 to 3 times a week  4 or more times a week |
| Namibia | How many days have you consumed alcohol in the past two weeks?  None  1-2  3-4  5+ |
| South Africa | None  Once a month or less  2- 4 times a month  2-3 times a week  4 or more times a week |
| United Republic of Tanzania | None  Sporadic  Monthly  Weekly  Daily |

Other countries did not collect data on alcohol drinking.

## **Figure S1. Results of the updated literature search**

##

**
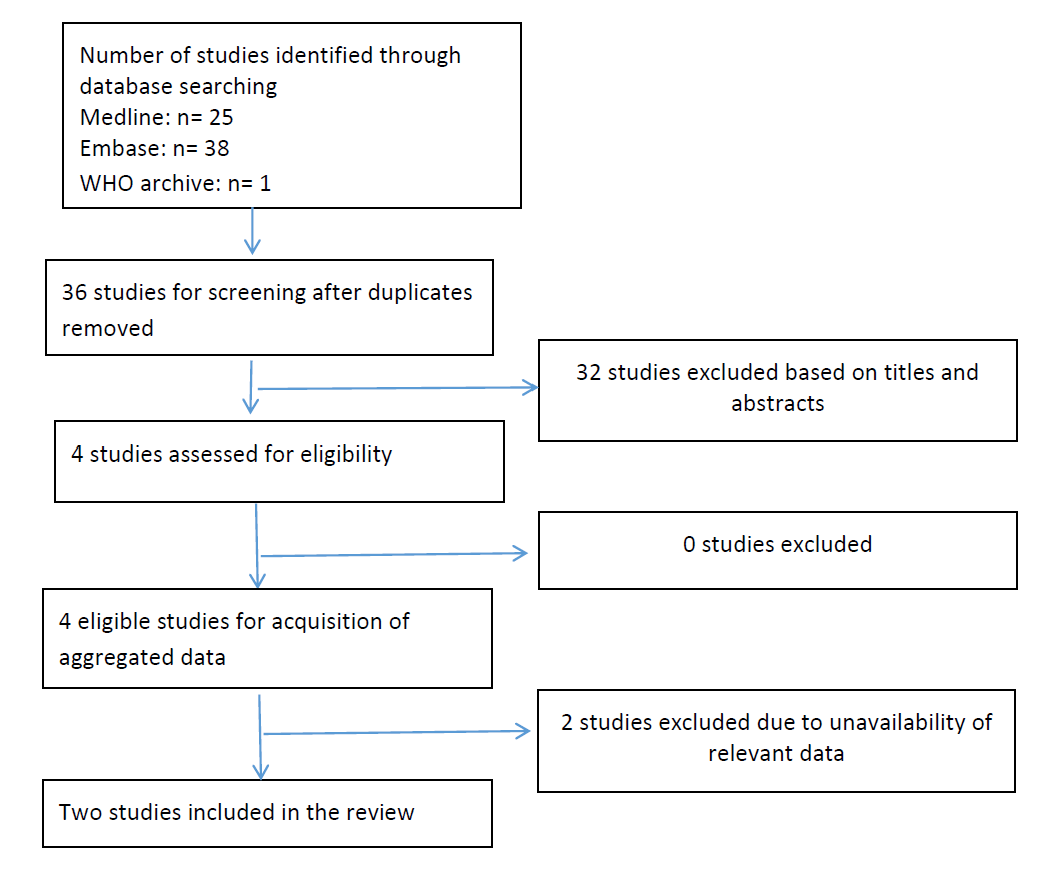
**

WHO: world health organization

## **Table S2-1. Characteristics of included national TB prevalence surveys**

|  | Bangladesh | Eswatini | Gambia | Ghana | Indonesia | Lesotho | Malawi | Mongolia |
| --- | --- | --- | --- | --- | --- | --- | --- | --- |
| Year | 2015-2016 | 2018 | 2012 | 2013 | 2013-2014 | 2019 | 2013-2014 | 2014-2015 |
| n | 98710 | 24358 | 43100 | 61726 | 67944 | 21719 | 31579 | 50309 |
| Age (mean (SD)) | 36.21 (16.32) | 36.04 (17.58) | 33.12 (16.52) | 37.77 (17.49) | 39.22 (16.22) | 41.07 (19.45) | 34.65 (16.86) | 40.70 (15.97) |
| Gender (%) |  |  |  |  |  |  |  |  |
| Female | 54345 ( 55.1) | 14419 (59.2) | 25596 ( 59.4) | 37038 ( 60.0) | 36312 ( 53.4) | 13122 ( 60.4) | 18480 ( 58.5) | 30239 (60.1) |
| Male | 44365 ( 44.9) | 9939 (40.8) | 17504 ( 40.6) | 24688 ( 40.0) | 31632 ( 46.6) | 8597 ( 39.6) | 13099 ( 41.5) | 20070 (39.9) |
| NA | 0 ( 0.0) | 0 ( 0.0) | 0 ( 0.0) | 0 ( 0.0) | 0 ( 0.0) | 0 ( 0.0) | 0 ( 0.0) | 0 ( 0.0) |
| TB (%) |  |  |  |  |  |  |  |  |
| No | 98263 ( 99.5) | 23261 (95.5) | 42511 ( 98.6) | 61339 ( 99.4) | 67199 ( 98.9) | 20951 ( 96.5) | 31331 ( 99.2) | 49248 (97.9) |
| Yes | 278 ( 0.3) | 70 ( 0.3) | 77 ( 0.2) | 202 ( 0.3) | 426 ( 0.6) | 132 ( 0.6) | 132 ( 0.4) | 248 ( 0.5) |
| NA | 169 ( 0.2) | 1027 ( 4.2) | 512 ( 1.2) | 185 ( 0.3) | 319 ( 0.5) | 636 ( 2.9) | 116 ( 0.4) | 813 ( 1.6) |
| Diabetes (%) |  |  |  |  |  |  |  |  |
| No | 0 ( 0.0) | 5774 (23.7) | 0 ( 0.0) | 2528 ( 4.1) | 66290 ( 97.6) | 0 ( 0.0) | 0 ( 0.0) | 49070 (97.5) |
| Yes | 0 ( 0.0) | 231 ( 0.9) | 0 ( 0.0) | 103 ( 0.2) | 1654 ( 2.4) | 0 ( 0.0) | 0 ( 0.0) | 1235 ( 2.5) |
| NA | 98710 (100.0) | 18353 (75.3) | 43100 (100.0) | 59095 ( 95.7) | 0 ( 0.0) | 21719 (100.0) | 31579 (100.0) | 4 ( 0.0) |
| HIV (%) |  |  |  |  |  |  |  |  |
| Negative | 0 ( 0.0) | 3741 (15.4) | 0 ( 0.0) | 0 ( 0.0) | 0 ( 0.0) | 13116 ( 60.4) | 17863 ( 56.6) | 50231 (99.8) |
| Positive | 0 ( 0.0) | 1674 ( 6.9) | 0 ( 0.0) | 0 ( 0.0) | 0 ( 0.0) | 3915 ( 18.0) | 1840 ( 5.8) | 75 ( 0.1) |
| NA | 98710 (100.0) | 18943 (77.8) | 43100 (100.0) | 61726 (100.0) | 67944 (100.0) | 4688 ( 21.6) | 11876 ( 37.6) | 3 ( 0.0) |
| Alcohol use (%) |  |  |  |  |  |  |  |  |
| None | 0 ( 0.0) | 6632 (27.2) | 42655 ( 99.0) | 1856 ( 3.0) | 0 ( 0.0) | 0 ( 0.0) | 0 ( 0.0) | 27149 (54.0) |
| Once a week or less | 0 ( 0.0) | 991 ( 4.1) | 371 ( 0.9) | 570 ( 0.9) | 0 ( 0.0) | 0 ( 0.0) | 0 ( 0.0) | 22616 (45.0) |
| Twice a week or more | 0 ( 0.0) | 427 ( 1.8) | 59 ( 0.1) | 393 ( 0.6) | 0 ( 0.0) | 0 ( 0.0) | 0 ( 0.0) | 129 ( 0.3) |
| NA | 98710 (100.0) | 16308 (67.0) | 15 ( 0.0) | 58907 ( 95.4) | 67944 (100.0) | 21719 (100.0) | 31579 (100.0) | 415 ( 0.8) |
| Smoking (%) |  |  |  |  |  |  |  |  |
| Never | 71353 ( 72.3) | 6284 (25.8) | 36152 ( 83.9) | 2491 ( 4.0) | 0 ( 0.0) | 13946 ( 64.2) | 26512 ( 84.0) | 36299 (72.2) |
| Past smoking | 5941 ( 6.0) | 578 ( 2.4) | 1987 ( 4.6) | 176 ( 0.3) | 0 ( 0.0) | 1854 ( 8.5) | 2036 ( 6.4) | 1506 ( 3.0) |
| Current smoking | 21416 ( 21.7) | 667 ( 2.7) | 4961 ( 11.5) | 152 ( 0.2) | 23025 ( 33.9) | 5848 ( 26.9) | 3031 ( 9.6) | 12291 (24.4) |
| Non-current smoker (no data on past smoking) | 0 ( 0.0) | 576 ( 2.4) | 0 ( 0.0) | 0 ( 0.0) | 44919 ( 66.1) | 0 ( 0.0) | 0 ( 0.0) | 0 ( 0.0) |
| NA | 0 ( 0.0) | 16253 (66.7) | 0 ( 0.0) | 58907 ( 95.4) | 0 ( 0.0) | 71 ( 0.3) | 0 ( 0.0) | 213 ( 0.4) |
| Any TB symptoms (%) |  |  |  |  |  |  |  |  |
| No | 71827 ( 72.8) | 21951 (90.1) | 27444 ( 63.7) | 130 ( 0.2) | 31572 ( 46.5) | 17355 ( 79.9) | 28890 ( 91.5) | 37588 (74.7) |
| Yes | 26883 ( 27.2) | 2393 ( 9.8) | 15656 ( 36.3) | 5822 ( 9.4) | 36372 ( 53.5) | 4348 ( 20.0) | 2689 ( 8.5) | 12481 (24.8) |
| NA | 0 ( 0.0) | 14 ( 0.1) | 0 ( 0.0) | 55774 ( 90.4) | 0 ( 0.0) | 16 ( 0.1) | 0 ( 0.0) | 240 ( 0.5) |
| Past history of TB (%) |  |  |  |  |  |  |  |  |
| No | 96802 ( 98.1) | 22512 (92.4) | 42612 ( 98.9) | 61285 ( 99.3) | 65753 ( 96.8) | 19776 ( 91.1) | 3186 ( 10.1) | 48308 (96.0) |
| Yes | 1908 ( 1.9) | 1793 ( 7.4) | 488 ( 1.1) | 359 ( 0.6) | 2191 ( 3.2) | 1943 ( 8.9) | 250 ( 0.8) | 2001 ( 4.0) |
| NA | 0 ( 0.0) | 53 ( 0.2) | 0 ( 0.0) | 82 ( 0.1) | 0 ( 0.0) | 0 ( 0.0) | 28143 ( 89.1) | 0 ( 0.0) |

TB: tuberculosis; SD: standard deviation; HIV: human immunodeficiency virus

## **Table S2-2. Characteristics of included national TB prevalence surveys**

|  | Mozambique | Namibia | Nigeria | Philippines | South Africa | United Republic of Tanzania | Uganda | Viet Nam |
| --- | --- | --- | --- | --- | --- | --- | --- | --- |
| Year | 2017 | 2017 | 2012 | 2016 | 2017-2019 | 2012 | 2014-2015 | 2017 |
| n | 32445 | 29495 | 12999 | 46689 | 35191 | 50447 | 41154 | 61763 |
| Age (mean (SD)) | 33.90 (16.70) | 37.77 (17.60) | 39.19 (17.68) | 39.47 (17.62) | 40.55 (18.33) | 38.14 (17.84) | 33.50 (15.76) | 46.55 (16.96) |
| Gender (%) |  |  |  |  |  |  |  |  |
| Female | 18444 ( 56.8) | 16900 (57.3) | 7631 ( 58.7) | 25796 ( 55.3) | 21803 (62.0) | 29701 (58.9) | 23669 ( 57.5) | 34613 ( 56.0) |
| Male | 14001 ( 43.2) | 12595 (42.7) | 5368 ( 41.3) | 20893 ( 44.7) | 13388 (38.0) | 20735 (41.1) | 17485 ( 42.5) | 27150 ( 44.0) |
| NA | 0 ( 0.0) | 0 ( 0.0) | 0 ( 0.0) | 0 ( 0.0) | 0 ( 0.0) | 11 ( 0.0) | 0 ( 0.0) | 0 ( 0.0) |
| TB (%) |  |  |  |  |  |  |  |  |
| No | 29608 ( 91.3) | 27802 (94.3) | 12467 ( 95.9) | 43867 ( 94.0) | 33466 (95.1) | 49326 (97.8) | 40691 ( 98.9) | 61108 ( 98.9) |
| Yes | 89 ( 0.3) | 119 ( 0.4) | 66 ( 0.5) | 466 ( 1.0) | 234 ( 0.7) | 159 ( 0.3) | 160 ( 0.4) | 221 ( 0.4) |
| NA | 2748 ( 8.5) | 1574 ( 5.3) | 466 ( 3.6) | 2356 ( 5.0) | 1491 ( 4.2) | 962 ( 1.9) | 303 ( 0.7) | 434 ( 0.7) |
| Diabetes (%) |  |  |  |  |  |  |  |  |
| No | 0 ( 0.0) | 11714 (39.7) | 0 ( 0.0) | 44823 ( 96.0) | 32867 (93.4) | 5929 (11.8) | 0 ( 0.0) | 4154 ( 6.7) |
| Yes | 0 ( 0.0) | 183 ( 0.6) | 0 ( 0.0) | 1866 ( 4.0) | 1784 ( 5.1) | 61 ( 0.1) | 0 ( 0.0) | 376 ( 0.6) |
| NA | 32445 (100.0) | 17598 (59.7) | 12999 (100.0) | 0 ( 0.0) | 540 ( 1.5) | 44457 (88.1) | 41154 (100.0) | 57233 ( 92.7) |
| HIV (%) |  |  |  |  |  |  |  |  |
| Negative | 19879 ( 61.3) | 21053 (71.4) | 0 ( 0.0) | 0 ( 0.0) | 21800 (61.9) | 5695 (11.3) | 3972 ( 9.7) | 0 ( 0.0) |
| Positive | 2966 ( 9.1) | 3338 (11.3) | 0 ( 0.0) | 0 ( 0.0) | 4606 (13.1) | 307 ( 0.6) | 422 ( 1.0) | 0 ( 0.0) |
| NA | 9600 ( 29.6) | 5104 (17.3) | 12999 (100.0) | 46689 (100.0) | 8785 (25.0) | 44445 (88.1) | 36760 ( 89.3) | 61763 (100.0) |
| Alcohol use (%) |  |  |  |  |  |  |  |  |
| None | 10248 ( 31.6) | 7086 (24.0) | 0 ( 0.0) | 0 ( 0.0) | 23323 (66.3) | 3744 ( 7.4) | 0 ( 0.0) | 0 ( 0.0) |
| Once a week or less | 1938 ( 6.0) | 2028 ( 6.9) | 0 ( 0.0) | 0 ( 0.0) | 9858 (28.0) | 1166 ( 2.3) | 0 ( 0.0) | 0 ( 0.0) |
| Twice a week or more | 362 ( 1.1) | 1399 ( 4.7) | 0 ( 0.0) | 0 ( 0.0) | 2010 ( 5.7) | 1085 ( 2.2) | 0 ( 0.0) | 0 ( 0.0) |
| NA | 19897 ( 61.3) | 18982 (64.4) | 44186 (100.0) | 46689 (100.0) | 0 ( 0.0) | 44452 (88.1) | 41154 (100.0) | 61763 (100.0) |
| Smoking (%) |  |  |  |  |  |  |  |  |
| Never | 0 ( 0.0) | 0 ( 0.0) | 39950 ( 90.4) | 28128 ( 60.2) | 0 ( 0.0) | 4640 ( 9.2) | 35412 ( 86.0) | 2453 ( 4.0) |
| Past smoking | 0 ( 0.0) | 0 ( 0.0) | 2096 ( 4.7) | 7637 ( 16.4) | 0 ( 0.0) | 476 ( 0.9) | 2715 ( 6.6) | 620 ( 1.0) |
| Current smoking | 1337 ( 4.1) | 2196 ( 7.4) | 2139 ( 4.8) | 10749 ( 23.0) | 9367 (26.6) | 875 ( 1.7) | 3020 ( 7.3) | 1459 ( 2.4) |
| Non-current smoker (no data on past smoking) | 11183 ( 34.5) | 9916 (33.6) | 0 ( 0.0) | 0 ( 0.0) | 25750 (73.2) | 11 ( 0.0) | 0 ( 0.0) | 0 ( 0.0) |
| NA | 19925 ( 61.4) | 17383 (58.9) | 1 ( 0.0) | 175 ( 0.4) | 74 ( 0.2) | 44445 (88.1) | 7 ( 0.0) | 57231 ( 92.7) |
| Any TB symptoms (%) |  |  |  |  |  |  |  |  |
| No | 24072 ( 74.2) | 21072 (71.4) | 0 ( 0.0) | 25608 ( 54.8) | 29589 (84.1) | 0 ( 0.0) | 27941 ( 67.9) | 1329 ( 2.2) |
| Yes | 7394 ( 22.8) | 8422 (28.6) | 3928 ( 30.2) | 19943 ( 42.7) | 5168 (14.7) | 1497 ( 3.0) | 13213 ( 32.1) | 11402 ( 18.5) |
| NA | 979 ( 3.0) | 1 ( 0.0) | 9071 ( 69.8) | 1138 ( 2.4) | 434 ( 1.2) | 48950 (97.0) | 0 ( 0.0) | 49032 ( 79.4) |
| Past history of TB (%) |  |  |  |  |  |  |  |  |
| No | 31298 ( 96.5) | 26515 (89.9) | 12815 ( 98.6) | 43993 ( 94.2) | 32099 (91.2) | 49192 (97.5) | 40342 ( 98.0) | 60371 ( 97.7) |
| Yes | 1064 ( 3.3) | 2979 (10.1) | 184 ( 1.4) | 2615 ( 5.6) | 2964 ( 8.4) | 740 ( 1.5) | 812 ( 2.0) | 1130 ( 1.8) |
| NA | 83 ( 0.3) | 1 ( 0.0) | 0 ( 0.0) | 81 ( 0.2) | 128 ( 0.4) | 515 ( 1.0) | 0 ( 0.0) | 262 ( 0.4) |

TB: tuberculosis; SD: standard deviation; HIV: human immunodeficiency virus

##

## **Table S3. Quality of individual surveys**

| Survey | Selection | Measurement of the outcome | | | | Measurement of the exposure | Missing data | |
| --- | --- | --- | --- | --- | --- | --- | --- | --- |
|  | # participated/# eligible (%) | Symptom screening criteria | Chest x-ray criteria | | Diagnostic method | Diagnosis of diabetes | All four symptoms collected?**** | NCDs data sought in all participants? |
| Bangladesh | 98710/108834 (90.7) | Scoring based on cough, haemoptysis, weight loss, fever, and/or night sweats | | Any lung abnormality | Smear, culture, and Xpert | NA | Yes | Yes |
| Eswatini | 24358/NA (NA) | Cough of any duration, fever for ≥ 2 weeks, unexplained weight loss ≥ 2 weeks, and/or night sweats ≥ 2 weeks | | Any lung abnormality | Xpert. Culture on Xpert positive samples | Self-report | Yes | In participants eligible for sputum collection and a randomly selected subset of the others. |
| Gambia | 43100/55832 (77.2) | Cough ≥ 2 weeks,  Cough < 2 weeks with ≥ 2 other TB symptoms*, or  No cough with ≥ 3 other TB symptoms* | | Any lung or mediastinum abnormality | Smear and Culture. Xpert for survey TB cases | NA | Yes | Yes |
| Ghana | 61726/67757 (91.1) | Cough ≥ 2 weeks | | Any lung abnormality | Smear and Culture. Xpert on smear+ samples, and if cultures contaminated | Self-report | Fever, weight loss, and night sweats collected only in participants who had cough > 2 weeks, prevalent TB, or TB treatment | In participants who had cough ≥ 2 weeks, TB diagnosis, or treatment history |
| Indonesia | 67944/76576 (88.7) | Cough ≥ 2 weeks and/or haemoptysis | | Any lung or pleura abnormality | Smear and Culture. Xpert on smear+ and non-conclusive culture samples | Self-report | Yes | Yes |
| Lesotho | 21719/26857 (80.9) | Cough ≥ 2 weeks, fever, weight loss, and/or night sweats | | Any lung abnormality | Xpert and culture | NA | Yes | Yes |
| Malawi | 31579/39026 (80.9) | Any symptoms** ≥ 1 week | | Any lung abnormality | Smear and Culture. Xpert on smear+ or if culture contaminated | NA | Yes | Yes |
| Mongolia | 50309/60031 (83.8) | Cough ≥ 2 weeks | | Any lung abnormality | Smear and Culture, Xpert on smear+ samples | Self-report | Yes | Yes |
| Mozambique | 32445/43442 (74.7) | Cough ≥2 weeks, blood in sputum, and/or any cough with one of the five symptoms/signs for ≥ 2 weeks*** | | Any lung or mediastinum abnormality or CAD4TB score ≥ 40 | Smear, Xpert, and Culture | NA | Yes | In participants eligible for sputum collection and a randomly selected subset of the others. |
| Namibia | 29495/38353 (76.9) | Cough, night sweats, fever, and/or weight loss | | Any lung abnormality or CAD4TB score ≥60 | Smear, Xpert, and Culture | Self-report | Yes | In participants eligible for sputum collection and a randomly selected subset of the others. |
| Nigeria | 44186/77707 (56.8) | Cough ≥ 2 weeks | | Any lung abnormality | Smear, culture, and Xpert | NA | Night sweats not collected. | Yes |
| Philippines | 35191/53250 (66.1) | Cough ≥2 weeks, blood in the sputum, and/or haemoptysis | | Any lung abnormality | Smear and Culture | Self-report | Yes | Yes |
| South Africa | 46689/61466 (76) | Any cough, fever, night sweats, and/or weight loss | | Any TB suggestive abnormality | Xpert Ultra and culture | Self-report | Yes | Yes |
| United Republic of Tanzania | 50447/65664 (76.8) | Cough ≥ 2 weeks, haemoptysis, fever ≥2 weeks, weight loss, and/or night sweats | | Any lung (or mediastinum) abnormality | Smear, culture, and Xpert  A concern raised about the validity of the number of bacteriologically positive cases. | Self-report | Current cough not collected. | In participants eligible for sputum submission |
| Uganda | 41154/45293 (90.9) | Cough ≥ 2 weeks | | Any lung abnormality | Smear and culture. Xpert on smear+ samples | NA | Yes | Yes |
| Viet Nam | 61763/87207 (70.8) | Productive cough ≥ 2 weeks | | Any lung abnormality | Smear and culture | Self-report | Weight loss, fever, and night sweats were asked only in participants who were eligible for sputum submission. | In participants eligible for sputum submission |

*Chest pain, night sweats, shortness of breath, loss of appetite, weight loss, fever, haemoptysis.

**Cough, sputum production, haemoptysis, chest pain, weight loss, night sweats, fatigue, fever, and shortness of breath.

***Chest pain, unexplained fever, night sweats, weight loss, and low mid-upper arm circumference

**** Current cough, fever, night sweats, and weight loss.

TB: tuberculosis; non-communicable diseases (NCDs)

##

## **Figure S2.Proportion of missing data by variable and by survey**


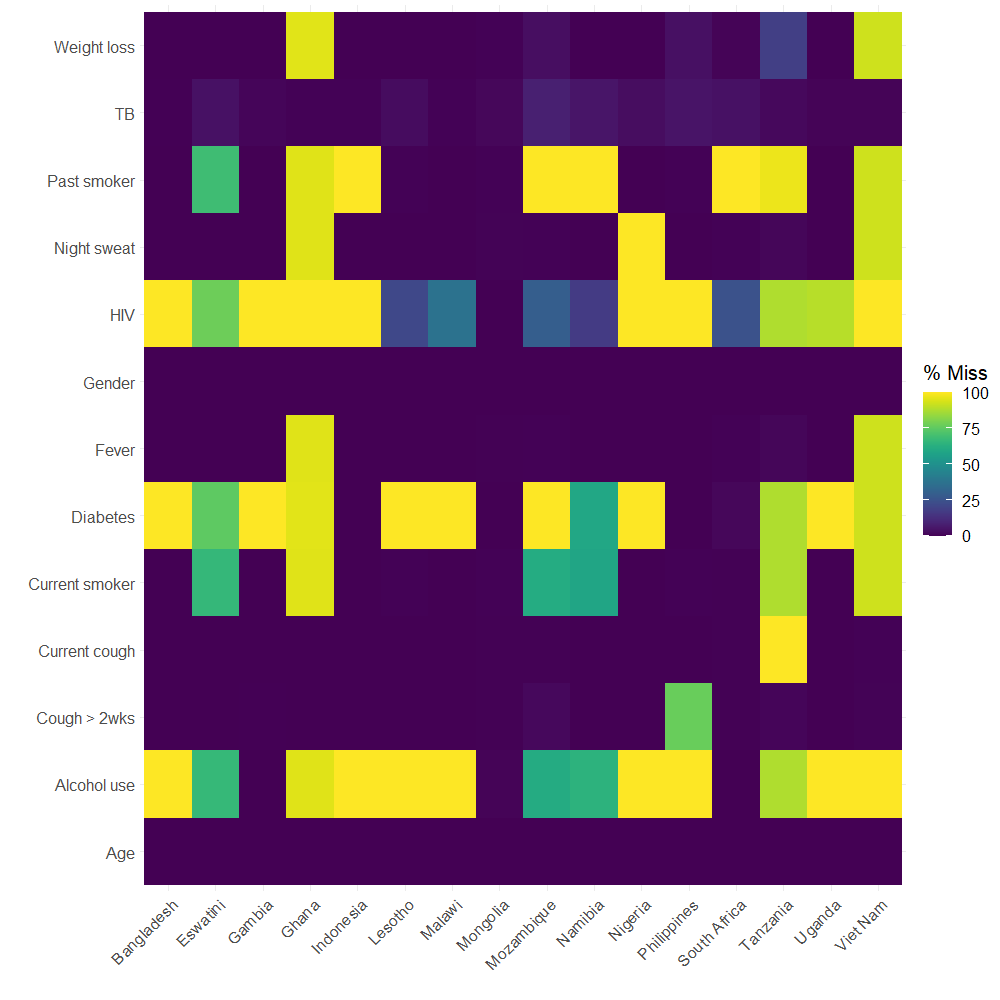


TB: tuberculosis; HIV: human immunodeficiency virus

## **Table S4. Studies found through the updated literature search**

| **Survey** | **Design** | **Criteria for sputum collection** | **Diagnostic methods** | **NCD-related variables** | **Findings** |
| --- | --- | --- | --- | --- | --- |
| Indian Council of Medical Research, 2022 | National survey during 2019-2021, including 322480 adults. | - TB symptoms (Cough for ≥2 weeks, fever for ≥ 2 weeks, significant weight loss, blood in sputum any time during last 6 months, chest pain in the previous one month) - History of TB - Chest X-ray findings suggestive of TB | Smear, culture, and PCR. | Diabetes (undefined), smoking, alcohol use, and BMI | The survey report stated that “individuals with abnormal chest X-ray, BMI < 18.5, more than any one symptom suggestive of TB, diabetes, past TB patients, alcohol consumption and smoking had higher likelihood to be TB positive”. No further details are reported including the analysis method. |
| Dolla et al, 2022 | A subnational survey in Thirvallur  District, India during 2015-2018. Included 69054 adults. | - TB symptoms (Cough for ≥ 3 weeks, chest pain for one month, coughing up blood sputum, weakness or fatigue, weight loss, no appetite, chills, unexplained fever for 1 month, night sweat) - History of TB - X-ray findings suggestive of TB | Smear and culture. | Diabetes (undefined), smoking, and alcohol use | The study reported the findings from the step-wise logistic regressions. The final model included age, gender, BMI, diabetes, history of TB, current smoking, and current alcohol use.  Adjustd odds ratios (95% confidence interval):  Diabetes: 1.67 (0.99–2.83)  Current smoking: 1.26 (0.83–1.93)  Current alcohol use: 1.62 (1.14–2.32)  History of TB: 3.64 (2.20–6.02) |

TB: tuberculosis; BMI: body mass index; PCR: Polymerase chain reaction

## **Table S5. Crude prevalence of active TB and proportion of subclinical TB**

| Country | N | All TB (crude prevalence, %) | % Subclinical TB (95% CI) |
| --- | --- | --- | --- |
| Bangladesh | 98710 | 280 (0.28) | 40.3 (34.7-46.2) |
| Eswatini | 24358 | 74 (0.30) | 49.8 (38.1-61.5) |
| Gambia | 43100 | 86 (0.20) | 22.2 (14.0-33.3) |
| Ghana | 61726 | 204 (0.33) | 33.5 (24.5-43.8) |
| Indonesia | 67944 | 433 (0.64) | 15.1 (11.9-18.9) |
| Lesotho | 21719 | 140 (0.64) | 54.5 (45.8-62.9) |
| Malawi | 31579 | 134 (0.42) | 37.8 (29.9-46.4) |
| Mongolia | 50309 | 253 (0.50) | 51.6 (45.4-57.9) |
| Mozambique | 32445 | 108 (0.33) | 46.6 (36.1-57.3) |
| Namibia | 29495 | 145 (0.49) | 38.4 (30.3-47.1) |
| Nigeria | 44186 | 255 (0.58) | 19.9 (12.3-30.6) |
| Philippines | 46689 | 501 (1.07) | 27.3 (23.3-31.6) |
| South Africa | 35191 | 260 (0.74) | 56.7 (50.2-62.9) |
| United Republic of Tanzania | 50447 | 176 (0.35) | 35.5 (27.0-45.1) |
| Uganda | 41154 | 162 (0.39) | 23.6 (17.6-30.8) |
| Viet Nam | 61763 | 232 (0.38) | 41.8 (35.3-48.5) |

Note: Values are based on multiply imputed datasets.

TB: tuberculosis; CI: confidence interval

## **Figure S3. Past smoking and TB status by survey**


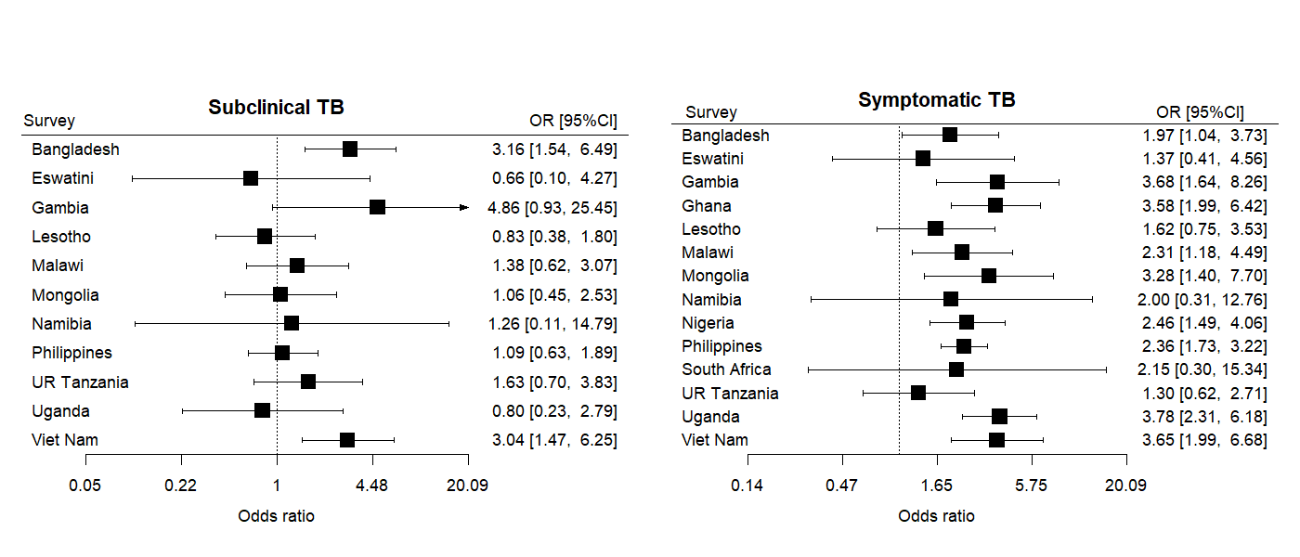


Subclinical TB: I-squared = 4.2% (95% CI 0-54.3), p = 0.4, tau^2^ = 0.13

Symptomatic TB: I-squared = 0% (95% CI 0-52.3), p = 0.61, tau^2^ = 0.0027

Surveys with large standard errors resulting in 95% confidence intervals ranging from 0 to infinity or for which the model failed to converge are excluded (Subclinical TB: Ghana, Indonesia, Mozambique, Nigeria, South Africa; symptomatic TB: Indonesia and Mozambique).

TB: tuberculosis; OR: odds ratio; CI: confidence intervals

## **Figure S4. Alcohol drinking and TB status by survey**


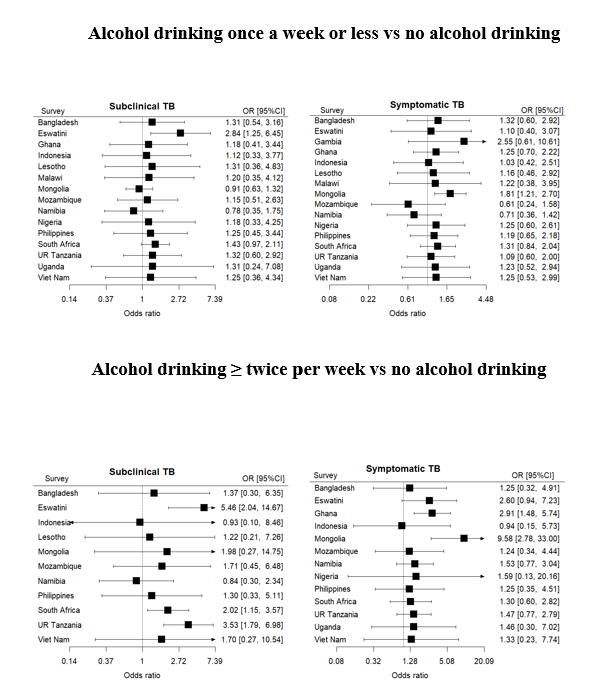


Note: Results of multivariable multiple regression models adjusted for age and gender by survey

Surveys with large standard errors resulting in 95% confidence intervals ranging from 0 to infinity or for which the model failed to converge are excluded (Subclinical TB: Gambia, Ghana, Malawi, Nigeria, Uganda; symptomatic TB: Gambia, Lesotho, and Malawi).

TB: tuberculosis; OR: odds ratio; CI: confidence intervals

**Alcohol drinking once a week or less vs no alcohol drinking**

Subclinical TB: I-squared = 0% (95% CI 0-52.3), p=0.91, tau^2^ = 0.01

Symptomatic TB: I-squared = 0% (95% CI 0-52.3), p=0.65, tau^2^ = 0.021

**Alcohol drinking once a week or less vs no alcohol drinking**

Subclinical TB: I-squared = 0% (95% CI 0-52.3), p=0.93, tau^2^ = 0.23

Symptomatic TB: I-squared = 0% (95% CI 0-52.3), p=0.65, tau^2^ = 0.021

## **Figure S5. HIV status and TB status by survey**


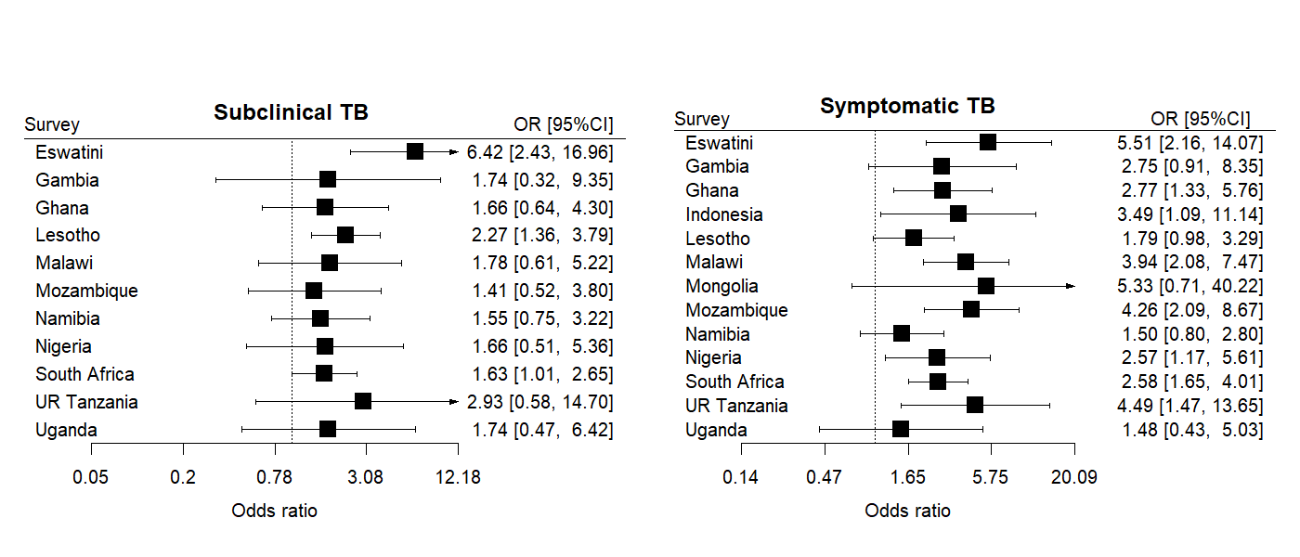


Note: Results of multivariable multiple regression models adjusted for age and gender by survey

Surveys with large standard errors resulting in 95% confidence intervals ranging from 0 to infinity or for which the model failed to converge are excluded (Subclinical TB: Indonesia, Lesotho, Mongolia, Philippines, Viet Nam; symptomatic TB: Lesotho, Philippines, Viet Nam).

TB: tuberculosis; OR: odds ratio; CI: confidence intervals

Subclinical TB: I-squared = 0% (95% CI 0-52.3), p = 0.93, tau^2^ = 0

Symptomatic TB: I-squared = 0% (95% CI 0-52.3), p = 0.62, tau^2^ = 0.027

## **Figure S6. Past history of TB and TB status by survey**


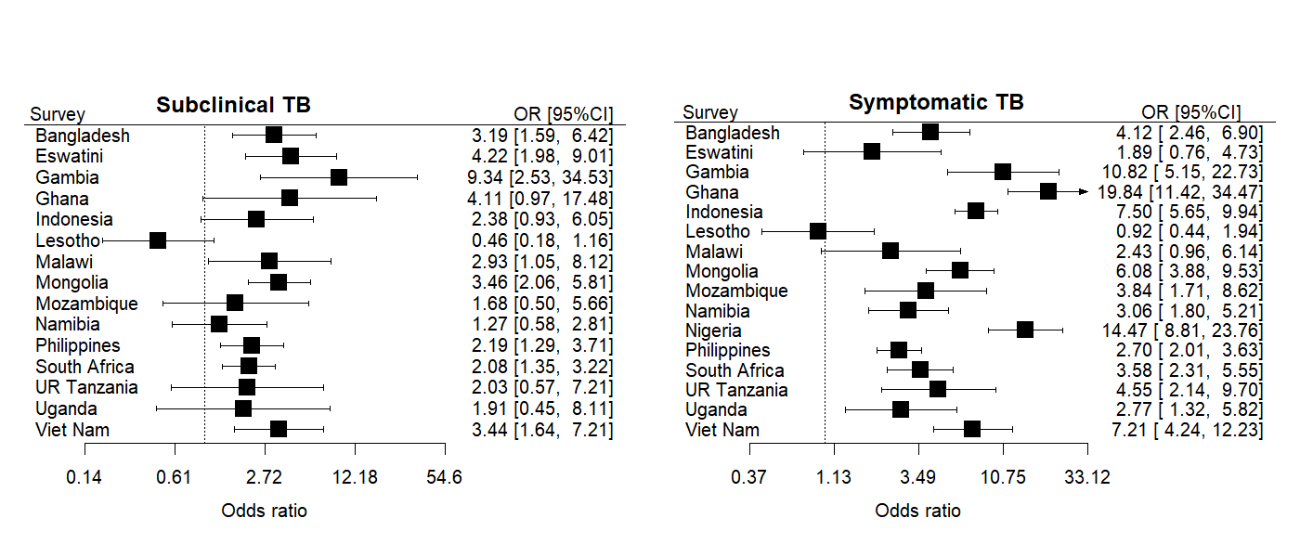


Note: Results of multivariable multiple regression models adjusted for age and gender by survey

Surveys with large standard errors resulting in 95% confidence intervals ranging from 0 to infinity or for which the model failed to converge are excluded (Subclinical TB: Nigeria; symptomatic TB).

TB: tuberculosis; OR: odds ratio: CI: confidence interval

Subclinical TB: I-squared = 42.74% (95% CI 0-68.3), p = 0.036, tau^2^ = 0.15

Symptomatic TB: I-squared = 86.14% (95% CI 79-90.8), p < 0.0001, tau^2^ = 0.5

## **Table S6. Associations between NCDs, NCD risk factors, and different manifestations of TB, adjusted for age and sex, in HIV-negative individuals**

|  | **All TB** | | **Subclinical TB** | | **Symptomatic TB** | |
| --- | --- | --- | --- | --- | --- | --- |
|  | Odds ratio (95% CI) | p-value | Odds ratio (95% CI) | p-value | Odds ratio (95% CI) | p-value |
| Current smoker vs non-current smoker | 1.72 (1.47-2.01) | <0.0001 | 1.76 (1.4-2.21) | <0.0001 | 1.68 (1.37-2.07) | <0.0001 |
| Past smoker vs never smoker | 1.65 (1.12-2.41) | 0.012 | 1.29 (0.78-2.14) | 0.32 | 1.97 (1.24-3.1) | 0.0044 |
| Alcohol drinking once a week or less vs no alcohol drinking | 1.13 (0.77-1.66) | 0.52 | 1.19 (0.82-1.74) | 0.35 | 1.08 (0.68-1.72) | 0.74 |
| Alcohol drinking ≥ twice per week vs no alcohol drinking | 1.4 (0.85-2.32) | 0.18 | 1.41 (0.83-2.41) | 0.2 | 1.38 (0.73-2.6) | 0.31 |
| Diabetes | 1.1 (0.71-1.69) | 0.67 | 1.1 (0.56-2.13) | 0.78 | 1.08 (0.61-1.91) | 0.79 |
| Past history of TB | 2.26 (1.83-2.79) | <0.0001 | 1.7 (1.22-2.37) | 0.0019 | 2.76 (2.13-3.58) | <0.0001 |

NCDs: non-communicable diseases; TB: tuberculosis; CI: confidence interval ; HIV : human immunodeficiency virus

## **Table S7. Sensitivity analysis using different categorisations of alcohol drinking**

| Definition | Outcome | Odds ratio (95% CI) | p-value |
| --- | --- | --- | --- |
| *Alcohol drinking once a week or less vs no alcohol drinking | All TB | 1.2 (0.91-1.58) | 0.18 |
|  | Subclinical TB | 1.33 (0.98-1.8) | 0.065 |
|  | Symptomatic TB | 1.14 (0.84-1.54) | 0.38 |
| *Alcohol drinking ≥ twice per week vs no alcohol drinking | All TB | 1.49 (0.64-3.48) | 0.34 |
|  | Subclinical TB | 1.59 (0.7-3.62) | 0.26 |
|  | Symptomatic TB | 1.43 (0.59-3.46) | 0.41 |
| Any alcohol drinking vs no drinking | All TB | 1.27 (0.85-1.89) | 0.23 |
|  | Subclinical TB | 1.39 (0.93-2.07) | 0.1 |
|  | Symptomatic TB | 1.21 (0.8-1.83) | 0.36 |
| Alcohol drinking ≥ twice per week vs less | All TB | 1.41 (0.64-3.1) | 0.37 |
|  | Subclinical TB | 1.46 (0.68-3.13) | 0.31 |
|  | Symptomatic TB | 1.38 (0.6-3.15) | 0.43 |

*Primary analysis

Estimates are adjusted for age and gender.

TB: tuberculosis; CI: confidence interval

## **Figure S7. Sensitivity analysis for the associations between all TB and predictors adjusted for age and gender**


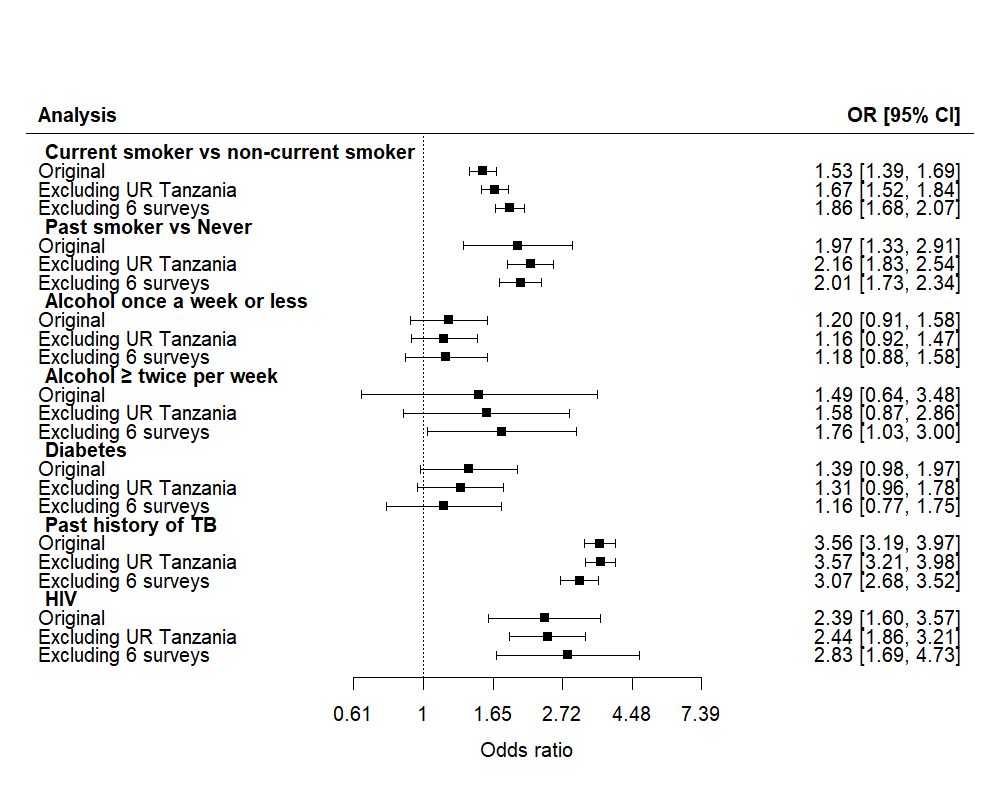


*Excluding 6 surveys (Eswatini, Ghana, Mozambique, Namibia, United Republic of Tanzania, Viet Nam) that collected NCD data only in a subset of participants.

TB: tuberculosis; HIV: human immunodeficiency virus; CI: confidence interval; OR: odds ratio; UR: United Republic of

## **Figure S8. Sensitivity analysis for the associations between subclinical TB and predictors adjusted for age and gender**


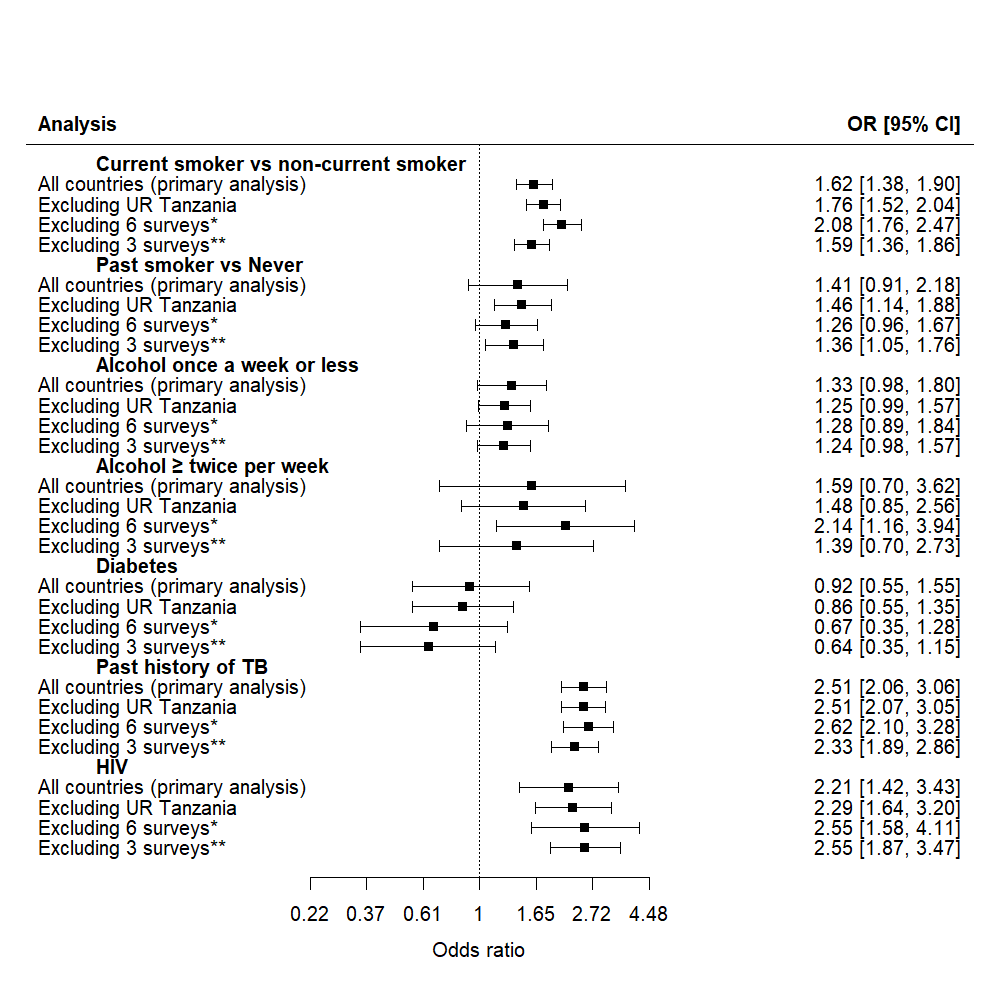


*Excluding 6 surveys (Eswatini, Ghana, Mozambique, Namibia, United Republic of Tanzania, Viet Nam) that collected NCD data only in a subset of participants.

** Excluding 3 surveys (Nigeria, United Republic of Tanzania, Viet Nam) that did not collect all four TB symptoms.

TB: tuberculosis; HIV: human immunodeficiency virus; CI: confidence interval; OR: odds ratio; UR: United Republic of

## **Figure S9. Sensitivity analysis for the associations between symptomatic TB and predictors adjusted for age and gender**


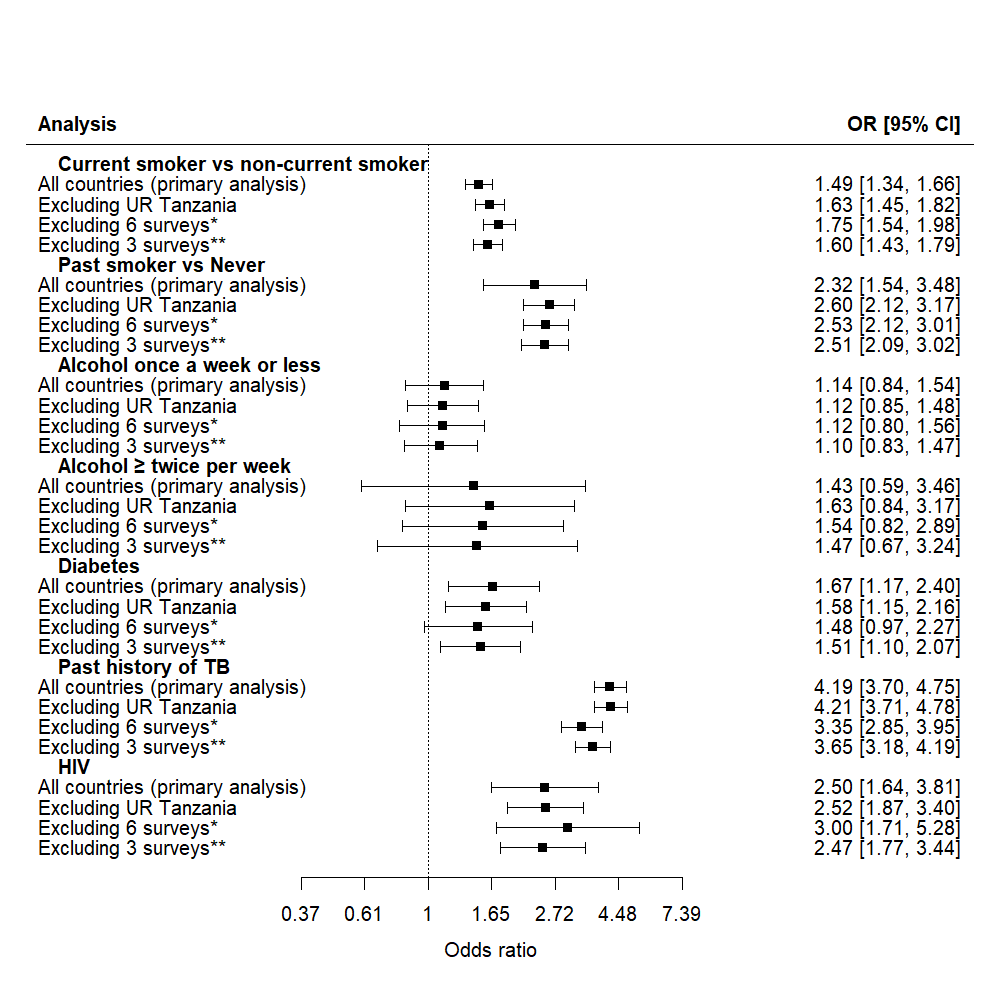


*Excluding 6 surveys (Eswatini, Ghana, Mozambique, Namibia, United Republic of Tanzania, Viet Nam) that collected NCD data only in a subset of participants.

** Excluding 3 surveys (Nigeria, United Republic of Tanzania, Viet Nam) that did not collect all four TB symptoms.

TB: tuberculosis; HIV: human immunodeficiency virus; CI: confidence interval; OR: odds ratio; UR: United Republic of

## **Table S8. Associations between NCDs, NCD risk factors, and different manifestations of TB, adjusted for age and sex, restricting to surveys with minimal missing data**

|  | **All TB** | | **Subclinical TB** | | **Symptomatic TB** | |
| --- | --- | --- | --- | --- | --- | --- |
|  | Odds ratio (95% CI) | p-value | Odds ratio (95% CI) | p-value | Odds ratio (95% CI) | p-value |
| Alcohol drinking once a week or less vs no alcohol drinking | 1.31 (1.08-1.59) | 0.0068 | 1.35 (1.04-1.77) | 0.026 | 1.27 (0.98-1.65) | 0.072 |
| Alcohol drinking ≥ twice per week vs no alcohol drinking | 1.73 (1.14-2.62) | 0.0099 | 2.19 (1.29-3.72) | 0.0035 | 1.3 (0.69-2.45) | 0.42 |
| Diabetes | 1.43 (1.12-1.81) | 0.0039 | 0.74 (0.42-1.28) | 0.28 | 1.78 (1.36-2.34) | <0.0001 |

NCDs: non-communicable diseases; TB: tuberculosis; CI: confidence interval

For alcohol drinking, the analysis was restricted to three surveys (Gambia, Mongolia, and South Africa), for diabetes, to four surveys (Indonesia, Mongolia, Philippines, and South Africa).

## **Figure S10. Sensitivity analysis-impact of misclassification of diabetic status**


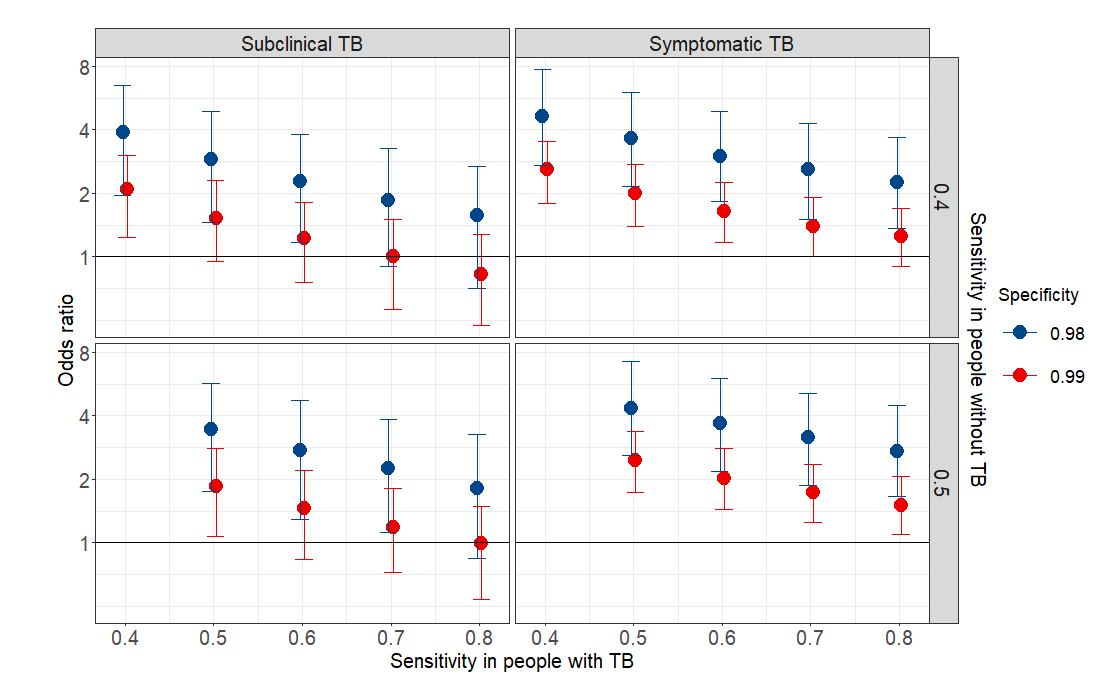


Odds ratios are adjusted for age and gender. Points and error bars indicate median and simulation intervals (2.5th and 97.5th percentiles of the estimates).

adjusted estimatesThe points error bars indicate that

Odds ratios in the analysis using original diabetic status:

Subclinical TB: 0.91 (95% CI 0.54-1.51)

Symptomatic TB: 1.65 (95% CI 1.16- 2.35)

The figure presents how the true association between diabetes and TB changes depending on the accuracy of self-reported diabetes. Overall, when the specificity or sensitivity is low, the association is more likely to be underestimated (i.e. higher odds ratio when misclassification is corrected). When diabetes is more likely to be diagnosed in people with TB than those without TB (i.e. higher sensitivity in people with diabetes), the association is more likely to be overestimated (i.e. lower odds ratio when misclassification is corrected). For symptomatic TB, the median estimates are consistently above one across different assumptions. For subclinical TB, the true association with diabetes tended to be positive in most scenarios in contrast to the association using the original diabetic status, especially if the specificity was 98%.

TB: tuberculosis; CI: confidence interval
